# Adsorption and Reactivity of Na^+^ Salt and Room-Temperature Ionic Liquid at Na Metal Surface

**DOI:** 10.1021/acs.jpcc.6c02391

**Published:** 2026-07-19

**Authors:** Ranjini Sarkar, Francesca Fasulo, Ana Belén Muñoz-García, Bernardino Tirri, Alfonso Amendola, Giacomo Melani, Simone Piccinin, Michele Pavone

**Affiliations:** † Department of Physics “E. Pancini”, 9307 University of Naples Federico II, Comp. Univ. Monte Sant’Angelo via Cintia 21, Napoli 80126, Italy; ‡ High Performance Computing Center of Excellence, DIT - Digital & Information Technology, 52845 Eni S.p.A., via Felice Maritano, 26, San Donato Milanese, Milan 20097, Italy; § Renewable, New Energies and Materials Science Research Center, Instituto Guido Donegani, 124223 Eni S.p.A., via Giacomo Fauser 4, Novara I-28100, Italy; ∥ 9327 CNR - Istituto Officina dei Materiali, c/o Sissa, via Bonomea 265, Trieste 34136, Italy; ⊥ Department of Chemical Sciences, University of Naples Federico II, Comp. Univ. Monte Sant’Angelo via Cintia 21, Napoli 80126, Italy

## Abstract

Sodium-ion batteries are cost-effective and sustainable alternatives to lithium-ion batteries. Recently, metal anode batteries have gained prominence over metal-ion systems due to their higher theoretical capacities. However, sodium metal batteries employing conventional organic electrolytes face persistent challenges, including dendrite formation and unstable solid-electrolyte interphase (SEI) layers. Thus, the choice of electrolyte plays a critical role in improving battery stability and performance. Ionic liquids (ILs) offer a promising pathway to improved performance owing to their high thermal and electrochemical stability and excellent ionic conductivity. In this work, we employ density functional theory (DFT) and *ab initio* molecular dynamics (AIMD) simulations to gain atomistic insights into SEI formation and stability by investigating the interactions of the IL containing *N*,*N*-methylpropylpyrrolidinium (PYR_13_^+^) cation, bis­(fluorosulfonyl)­amide (FSI^–^) and bis­(trifluoromethylsulfonyl)-amide (TFSI^–^) anions (PYR_13_FSI and PYR_13_TFSI), along with sodium salts NaFSI and NaTFSI, with the Na(110) metallic surface. Detailed geometric and electronic structure analyses capture the anode/electrolyte interfacial chemistry, encompassing both IL and salt interactions and their decomposition pathways to model the early stages of SEI formation. Our results show that FSI^–^ interacts more strongly than TFSI^–^ and undergoes spontaneous dissociation upon structural relaxation, whereas PYR_13_^+^ and TFSI^–^ remain intact. AIMD trajectories at 298 K over 5–10 ps reveal the formation of decomposition products, primarily NaF, which is known to contribute to SEI stabilization. Compared to FSI^–^, TFSI^–^ exhibits delayed decomposition, and PYR_13_^+^ cation remains intact throughout. Overall, these findings unravel the distinct interfacial behaviors of FSI^–^ and TFSI^–^ and highlight the critical role of anion chemistry in governing SEI formation and stability in sodium metal batteries.

## Graphical abstract

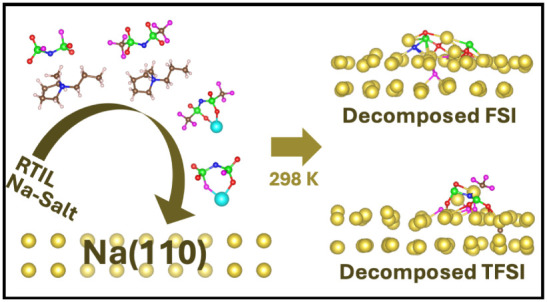

## 1 Introduction

Lithium-ion batteries (LIBs) have dominated rechargeable energy storage for decades; however, the increasing demand for large-scale and sustainable energy-storage technologies has enhanced the exploration of sodium-based batteries.^1,2^ Owing to its earth abundance, low cost, and chemical similarity to monovalent lithium, sodium offers a viable alternative that enables the partial transfer of established LIB design principles, particularly for grid-scale applications.^3,4^ Among sodium-based technologies, Na-metal batteries (NMBs) have attracted growing interest due to their potential for high energy density. The Na-metal anode provides a high theoretical specific capacity of 1166 mAh g^–1^ (compared to ∼300 mAh g^–1^ for Na-ion battery using hard carbon anodes)^5^ and a low redox potential (−2.71 V vs SHE). These intrinsic advantages position NMBs as promising candidates for next-generation energy-storage systems.^6–11^

A major bottleneck in NMBs is higher instability of the solid electrolyte interphase (SEI) compared to that in sodium-ion batteries.^12^ An ideal SEI is defined as a thin, ion-permeable, electronically insulating, mechanically robust, and self-passivating layer to suppress continuous electrolyte decomposition and increase battery life. The SEI, which forms spontaneously at Na metal/electrolyte interfaces, is typically irregular, brittle and unable to accommodate the large volume fluctuations during cycling. Repeated Na stripping and plating continuously disrupt the SEI, leading to poor kinetic performance and promoting moss-like dendritic growth.^13^ These dendrites accelerate parasitic reactions, produce electrically isolated “dead” sodium, reduce Coulombic efficiency, increase polarization, and may penetrate the separator, causing short circuits and thermal runaway.^14^ Plus, the conventional organic electrolytes pose additional safety risks at elevated temperatures due to their high flammability and vapor pressure.^15^ These challenges highlight the crucial role of electrolyte design for effective SEI stabilization.

In this framework, room-temperature ionic liquids (RTILs) have emerged as promising electrolytes for high-performance NMBs due to their high thermal and electrochemical stability and excellent ionic conductivity.^16^ In the ILs, often named “designer solvents”, an heterocyclic cation (imidazolium, pyridinium, pyrrolidinium, phosphonium, ammonium, etc.) is paired with inorganic anion (BF_4_^–^, PF_6_^–^, OAC^–^, FSI^–^, TFSI^–^, etc.), and the choice of cation–anion combination can tune the IL physicochemical properties.^16^ Among the anions, bis­(fluorosulfonyl)­amide (FSI^–^) and bis­(trifluoromethylsulfonyl)-amide (TFSI^–^) are the mostly used for electrolyte applications due the weakly coordination of cations, that enable higher ionic mobility.^17^

Imidazolium/FSI^–^ ionic liquids exhibit higher electrolyte stability than their TFSI^–^ counterparts due to suppressed anion decomposition. However, this enhanced stability can hinder the formation of a stable SEI on the Na-surface.^18^ In contrast, pyrrolidinium-based cations paired with either FSI^–^ or TFSI^–^ enable safer and more uniform sodium plating/stripping, resulting in more stable SEI layers.^19^ Seh et al. showed that the reductive decomposition of FSI^–^ and TFSI^–^ anions produces SEI enriched in inorganic species such as NaF and Na_2_O, with NaF confering high mechanical robustness to the SEI and effectively suppressing dendritic growth.^20^ Shkrob et al. designated FSI^–^ as “magic ion” due to its exceptional compatibility with Na metal, forming radical-derived passivation species that stabilize the Na surfaces.^21^ Furthermore, Rakov et al. showed that superconcentrated ionic-liquid electrolytes further enhance Na metal performance.^22^ A 1:1 PYR_13_FSI:NaFSI formulation improves Na cycling stability and enables stable Na plating/stripping for over 1000 h at 1 mA cm^–2^.^23^

Electrolytes based on Na salts (NaFSI or NaTFSI), either alone or combined with organic cations (salt-RTIL system Na­[FSI]+[C_3_C_1_pyr]­[FSI]), also strongly influence ion transport, solvation structure and interfacial reactivity. These salt-RTILs achieve favorable ionic conductivity, high electrochemical stability, and enhanced SEI formation, making them promising candidates for high-performance NMBs.^24^

Complementing the prior experimental investigations, density functional theory (DFT) and molecular dynamics (MD) simulations can provide detailed atomistic insight into electrolyte decomposition pathways and interfacial chemistry at metal surfaces.^25–27^ Previously, Zeng et al. proposed a “checkerboard” adsorption model for the ILs C_X_MPyr-FSI and TFSI (with varying alkyl chain length, “x”), providing a detailed quantum chemical description of the interface, albeit without explicitly addressing IL decomposition.^28^ Clarke-Hannaford et al. systematically investigated the adsorption and decomposition pathways of ILs NNBH_2_–FSI/TFSI and EtNH_3_–BF_4_ on Li(001) surface. Their studies revealed that FSI and TFSI anions decompose to form of LiF, Li_2_O, and Li_2_S as the primary decomposition products, while BF_4_ anion remained stable at room temperature owing to its relatively high activation energy of 0.21 eV. In both cases, the cations were found to remain intact, exhibiting only weak interaction with the Li surface.^29,30^ Along similar lines, Lu et al. compared the decomposition behavior of ILs containing hybrid anions, FTFSI and MCTFSI, paired with cation BMP on Li(001) surface.^31^

Despite these extensive *ab initio* studies on IL decomposition at lithium metal surfaces,^28–35^ comparable investigations on sodium remain limited. Moreover, previous studies have largely focused on the adsorption and decomposition of ILs alone. In realistic battery electrolytes, however, both ILs and salt species coexist and contribute to interfacial reactions. Therefore, explicit analyses of both ionic liquids and Na-salt dissociation pathways at Na metal surfaces are crucial to understand the SEI evolution in NMB devices.

To this aim, in this study, we combine DFT and ab initio molecular dynamics (AIMD) simulations to investigate the early stages of SEI formation on the Na(110) surface in IL-based electrolytes. AIMD is particularly important to capture finite-temperature effects and the dynamic behavior of ions at the interface, which are difficult to account for in static DFT calculations. We explicitly consider both key electrolyte components, the ionic-liquid ion pairs and the corresponding Na-salts, to examine their adsorption, structural rearrangements, and decomposition pathways at the sodium surface. For the ILs, we focus on the N,*N*-methylpropylpyrrolidinium (PYR_13_^+^) cation paired with FSI^–^ and TFSI^–^ anions, with particular attention to the differing behaviors of these anions. Our findings highlight that anion chemistry is the primary factor governing the initial stages of SEI formation on sodium metal. FSI^–^ decomposes rapidly to generate NaF, while TFSI^–^ undergoes slower and more complex fragmentation. These insights provide a molecular-level basis to design more stable and passivating electrolytes for high-performance sodium metal batteries.

## 2 Methods and Computational Details

The isolated cations (PYR_13_^+^ and Na^+^), anions (FSI^–^ and TFSI^–^), and their corresponding ion pairs (PYR_13_FSI, PYR_13_TFSI, NaFSI, and NaTFSI) were optimized in the gas phase within DFT employing Gaussian type orbital (GTO)-based triple-ζ 6–311+G­(d,p) basis set using the Gaussian16 package.^36^ Exchange-correlation effects were treated using both the generalized gradient approximation (PBE) and the hybrid B3LYP functional (denoted as GTO/PBE and GTO/B3LYP hereafter), with long-range dispersion interactions included via Grimme’s D3 correction.^37,38^ The stability of the optimized geometries is ensured by harmonic frequency calculations and the absence of imaginary frequencies.

To enable a consistent comparison between gas-phase and periodic descriptions, we also optimized the single ion pairs under periodic boundary conditions (PBC) by placing one molecule in a cubic box of 20 Å. Spin-polarized DFT calculations under PBC were carried out using the Vienna Ab initio Simulation Package (VASP, version 6.4.2).^39–42^ Core–valence interactions were treated using the projector-augmented wave (PAW)^37^ method with a plane-wave kinetic energy cutoff of 750 eV (denoted as PW/PBE hereafter). The resulting geometries of isolated ions and ion-pairs obtained with and without PBC are qualitatively similar, as discussed in detail in the following section. Charged systems were treated within periodic boundary conditions by adjusting the total number of electrons and finite-size electrostatic interactions between periodic images were corrected using the Makov-Payne correction. We also took into account dipole correction as implemented in VASP.^43^

Then, we investigated the interfacial interactions by explicitly adsorbing isolated ion pairs of the ionic liquids and salts on Na(110) surface, the most stable surface of sodium metal.^44^ A four-layer 4 × 4 Na slab (128 atoms) was constructed with lattice parameters a = 16.68 Å and b = 23.59 Å, and a vacuum spacing of 20 Å was introduced along the surface normal to avoid spurious interactions. Such supercells and vacuum spacing are sufficiently large to avoid intermolecular interactions between periodic images. The relative stability of the Na(110) surface compared to the (100) and (111) facets was also confirmed through surface energy calculations (Figure S1 and Table S1). Adsorbate molecules were initially positioned approximately 2.5 Å above the topmost Na layer. We used a Monkhorst–Pack k-point mesh of 4 × 4 × 1 based on convergence tests and Methfessel–Paxton smearing (first order, 0.1 eV) due to metallic sodium. We performed structural optimizations by relaxing the adsorbates and the top two layers of the Na slab until the residual forces and total energy changes were below 0.01 eV Å^–1^ and 10^–5^ eV, respectively. We used a denser 8 × 8 × 1 k-point grid for electronic structure analysis. Interfacial charge transfer was quantified via Bader charge analysis.^45^

The adsorption stability and decomposition dynamics of the electrolyte species at the Na(110) interface were further examined using AIMD simulations in VASP within the canonical ensemble (NVT). Temperature was maintained at 298 K using a Nosé-Hoover thermostat, and the equations of motion were integrated with a 1 fs time step over total simulation times of ∼10 ps. Structural and reaction pathways analysis were performed using VMD and VESTA visualization tools.^46,47^

## 3 Results and Discussion

### 3.1 Optimized Geometries and Electronic Structures of Electrolytes

First, we focused on single ion pairs of ionic liquids (PYR_13_FSI and PYR_13_TFSI) and sodium salts (NaFSI and NaTFSI). The geometries of the individual charged species (PYR_13_^+^, FSI^–^, and TFSI^–^), including *cis*/*trans* conformers (see Figure S2 and Table S2 in Supporting Information) show excellent consistency with previous studies, with the *trans* conformers being slightly more stable.^33^ Building on these reference structures of anions, we investigated the interionic interactions by constructing and optimizing multiple configurations of IL and salt-ion pairs under both gas-phase and periodic boundary conditions. For the most stable *trans* conformers of FSI^–^ and TFSI^–^, the ion-pair geometries and electronic properties are shown in Figure 1, while the corresponding *cis* conformers are reported in Figure S3 in Supporting Information.^27^ The interionic interaction energies of IL and salt ion-pairs (E_int,IL/salt_) were evaluated according to

1 $$E_{\mathrm{int},\mathrm{IL}/\mathrm{salt}}=E_{\mathrm{IL}/\mathrm{salt}}-\left(E_{{{\mathrm{PYR}}_{13}}^{+}/{\mathrm{Na}}^{+}}+E_{{\mathrm{FSI}}^{-}/{\mathrm{TFSI}}^{-}}\right)$$

where E_IL/salt_ are the total energies of the optimized IL/salt ion-pairs, $E_{{{\mathrm{PYR}}_{13}}^{+}/{\mathrm{Na}}^{+}}$ and $E_{{\mathrm{FSI}}^{-}/{\mathrm{TFSI}}^{-}}$ denote the total energies of isolated cations PYR_13_^+^ or Na^+^, and anions FSI^–^ or TFSI^–^, respectively.

**Figure 1:**
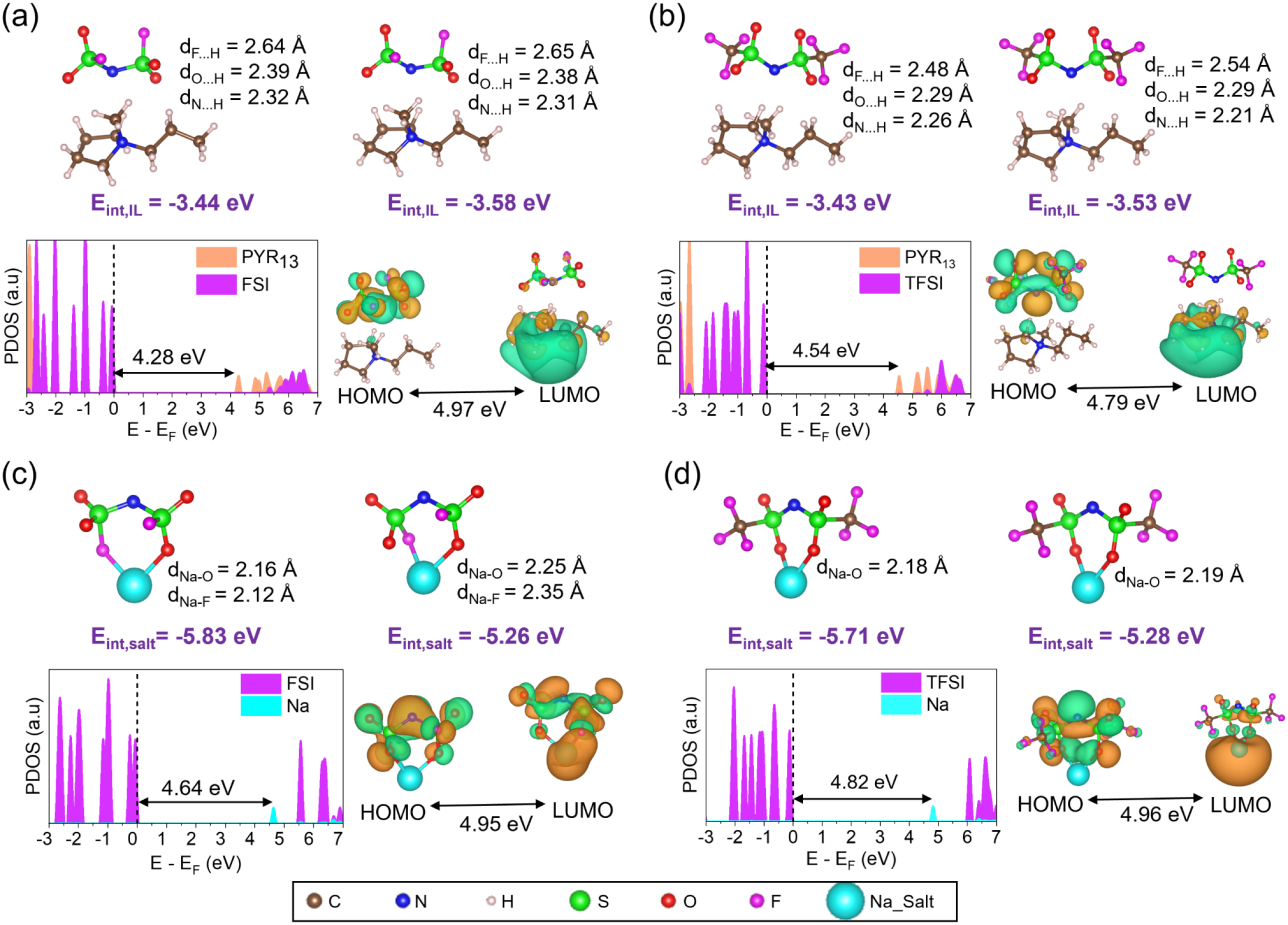
Minimum-energy structures and electronic structure of (a) PYR_13_FSI (*trans*-FSI), (b) PYR_13_TFSI (*trans*-TFSI), (c) NaFSI (*trans*-FSI) and (d) NaTFSI (*trans*-TFSI). For each panel, the left part is at the PW/PBE level of theory and right part at the GTO/PBE level. The minimum interionic bond lengths, anion/cation interaction energies (E_int,IL_ and E_int,salt_), PBC­[PBE] band gaps, and HOMO–LUMO gaps for all the ion-pairs are reported. Atomic color code: C (brown), N (blue), H (white), S (green), O (red), F (magenta), Na within salt (cyan). HOMO–LUMO isosurface value: 0.02 a.u, isosurface color code: positive (orange) and negative (green). PDOS color codes: PYR_13_ (orange), Na within salt (cyan), and FSI/TFSI (purple).

Although the *trans* conformers of FSI^–^ and TFSI^–^ are marginally more stable as isolated anions, no systematic dependence on anion conformation is observed in either the relative stability or the interionic interaction strengths of corresponding ion pairs. In contrast, the interactions between Na^+^ and the anions are consistently stronger than those between PYR_13_^+^ and the same anions. The calculated interaction energies are robust across all computational approaches, ranging from −3.58 to −3.39 eV for PYR_13_FSI, −3.64 to −3.43 eV for PYR_13_TFSI, −5.83 to −5.14 eV for NaFSI, and −5.71 to −5.28 eV for NaTFSI, in good agreement with previous reports.^31,48,49^ Notably, Lu et al. reported nearly identical interaction energies for *cis* and *trans* conformers in pyrrolidinium-based ionic liquids, with values of −3.280 and −3.281 eV for PYR_14_FSI and −3.295 and −3.293 eV for PYR_14_TFSI, respectively, further confirming the negligible influence of anion conformation on interionic binding.^31^

The nature of interionic interactions is illustrated through charge density difference (CDD) plots presented in Figure S5, where the positive and negative isosurfaces denote regions of charge accumulation and depletion, respectively. For the IL ion pairs, both positive and negative CDD isosurfaces are distributed across the anionic (FSI^–^ or TFSI^–^) and cationic (PYR_13_^+^) moieties. A net charge accumulation of ∼−0.06 a.u is observed on PYR_13_^+^, consistent with interaction motifs involving F···H, N···H, and O···H hydrogen bonds, with the bond distances provided in Figures 1, S2, and S3. In contrast, the salt ion pairs exhibit stronger and more localized charge accumulation and depletion at the cation (Na^+^) and anionic (FSI^–^ or TFSI^–^) sites, evidencing a pronounced charge-transfer of ∼−0.1 a.u. from the anion to cation. This substantial charge-transfer leads to the formation of Na–O and Na–F ionic interactions, resulting in more negative interaction energies (E_int,salt_) compared to those of the IL ion pairs (E_int,IL_), regardless of the anion configurations (*cis* or *trans*). Furthermore, no significant differences are observed in the intra- and interionic structural parameters among the most stable ion-pair geometries, as summarized in Table S3.

Figure 1 summarizes the electronic structure analysis based on projected density of states (PDOS) and gas-phase HOMO–LUMO orbitals (see Figure S3 for *cis* conformers). The PDOS results show that the highest occupied molecular orbital (HOMO) is dominated by anionic states, while the lowest unoccupied molecular orbital (LUMO) is primarily contributed by the cation, in agreement with the gas-phase molecular orbital analysis. Accordingly, as evident from the isosurface plots, the HOMO is largely localized on the anion and the LUMO on the cation for both ionic liquid and salt ion pairs, indicating donor–acceptor character within the ion pairs. Consistent with the interaction-energy trends, FSI- and TFSI-containing systems show nearly identical electronic gaps: PBC­[PBE] band gaps lie in the ranges 4.28–4.69 eV for ionic liquids and 4.63–4.82 eV for salts, consistent with HOMO–LUMO energy gaps (4.79–4.97 eV for ILs and 4.75–4.95 eV for salts). As expected from the well-known tendency of GGA functionals to underestimate band gaps,^50,51^ the PBE functional predicts smaller HOMO–LUMO gaps than the hybrid B3LYP functional (6.60–6.73 eV for IL and 6.32–6.75 eV for salts; see Figure S4 in SI). Nevertheless, both functionals yield the same qualitative description of the electronic structure of the isolated ion pairs, with the HOMO localized on the anion and the LUMO on the cation in all cases. The key electronic features governing the reduction and oxidation behavior of the electrolyte species are therefore consistently captured at the PBE level. Such results validate our approach for the following investigation of electrolyte molecules at Na interfaces at PBE level of theory, since this functional is furthermore the established method of choice for describing adsorption and reactivity at metallic surfaces.^25,27,29,31,52^

### 3.2 Adsorption of Electrolytes at Na(110) Surface

Starting from the most stable isolated ion-pair configurations (Figure 1), we investigated different adsorption modes of the electrolyte species on the Na(110) surface. The adsorption energy is evaluated as

2 $$E_{\mathrm{ads}}=E_{\mathrm{El}/\mathrm{Na}110}-\left(E_{\mathrm{El}}+E_{\mathrm{Na}110}\right)$$

where E_El/Na110_, E_El_, and E_Na110_ denote the total energies of the considered electrolytes (El), the ionic liquids PYR_13_-FSI/TFSI and salts Na-FSI/TFSI, adsorbed at Na(110) surface, the isolated electrolytes in its most stable conformation, and the bare Na(110) surface, respectively. Hereafter, we focus on the most stable *trans* anion FSI/TFSI configurations, as similar trends are observed for the corresponding *cis* configurations (data reported in Supporting Information).

For the ionic liquids PYR_13_FSI and PYR_13_TFSI, we explored several adsorption modes of the ionic pair on the Na(110) surface: (i) configurations with the cation or the anion is oriented vertically toward the surface, denoted cation-Na and anion-Na, respectively, (ii) configurations in which both ions interact simultaneously with the surface in distinct lateral arrangements, referred to as side-on modes (Figure S6 in SI).

We found that the cation-Na and anion-Na configurations display weaker adsorption, with *E*_ads_ values ranging from −0.96 to −0.21 eV, whereas the side-on configurations yield stronger binding with *E*_ads_ values between −1.44 and −1.18 eV (Figure 2 and Figure S6 in SI). For PYR_13_FSI an additional side-on configuration (denoted side-on 3: Diss­[FSI]) undergoes spontaneous dissociation upon adsorption on the Na (110) surface, leading to highly negative adsorption energies of −5.87 eV and −5.91 eV for the *trans* and *cis* FSI systems, respectively (in Figure 2c and Figure S7 in SI). No dissociation is observed for PYR or TFSI in any of the explored adsorption modes.

**Figure 2:**
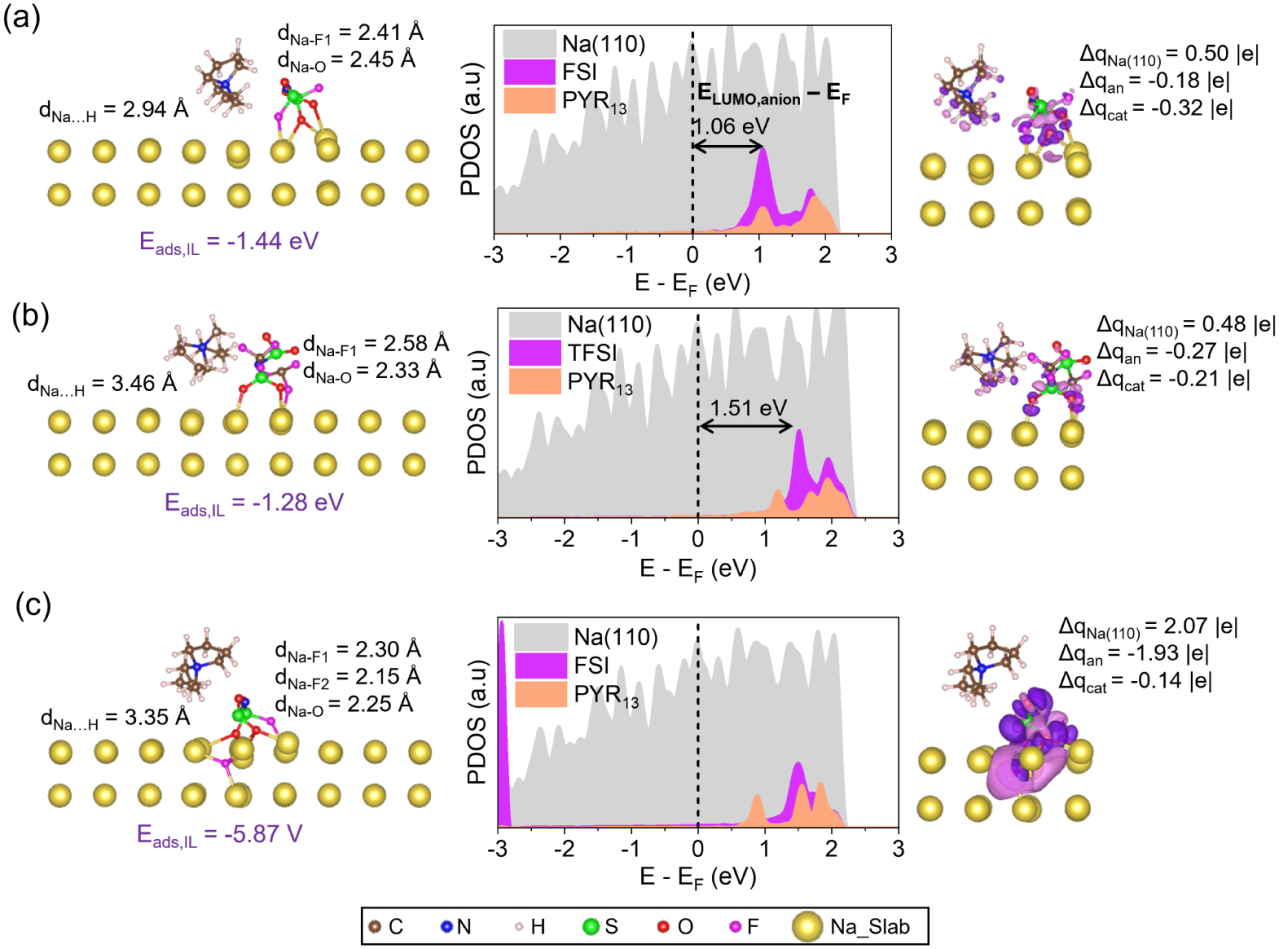
Minimum-energy structures of the most stable adsorption configurations of the ILs with the minimum Na-slab/cation (and anion) distances and IL adsorption energies *E*_ads,IL_ (left), PDOS plots (middle), and CDD isosurfaces with variations in Bader charge-transfer values (right) for (a) PYR_13_FSI (*trans*-FSI) with undissociated FSI, (b) PYR_13_TFSI (*trans*-TFSI), and (c) PYR_13_FSI (*trans*-FSI) with dissociated FSI adsorbed on Na(110) surface (Diss­[FSI]). F1 and F2 represent the undissociated and dissociated F atoms, respectively. Atomic color code: C (brown), N (blue), H (white), S (green), O (red), F (magenta), and Na within Na(110) slab (yellow). CDD isosurface value: 0.0005 a.u, isosurface color code: positive (blue) and negative (pink). PDOS color codes: Na(110) slab (gray), PYR_13_ (orange), and FSI/TFSI (purple). Theoretical level: PW/PBE.

In all cases, the structural analysis show that the PYR_13_^+^ cation interacts weakly with the surface, as indicated by long Na–H distances (2.94–3.98 Å), whereas the anions form stronger interfacial bonds. Undissociated FSI^–^ and TFSI^–^ exhibit ionic Na–F and Na–O interactions (Na–F: 2.30–2.91 Å; Na–O: 2.25–2.45 Å), while in Diss­[FSI] interaction mode, FSI^–^ dissociation leads to penetration of F into the Na layers and formation of shorter Na–F bonds (2.15–2.19 Å), consistent with the highly exothermic adsorption energies (Table S4). Further structural analyses are reported in Table S5 in SI, indicating similar hydrogen bond distances for the anion/cation interaction sites in the IL ion-pairs.

The variations in the Bader charges of the Na(110) surface (Δq_Na(110)_), IL-anion (Δq_an_), and IL-cation (Δq_cat_) are defined as the differences in the charge-transfer values in the adsorbed state with respect to their isolated counterparts. Bader charge analysis (see Table S4 in SI) reveals moderate charge transfer from Na(110) surface to the IL ion-pairs for undissociated adsorption modes, with a variation of 0.24–0.51 |e| and 0.28–0.55 |e| for PYR_13_FSI and PYR_13_TFSI, respectively, whereas FSI^–^ dissociation induces a dramatic increase in charge transfer (2.12 and 2.07 |e| for *cis* and *trans*, respectively), localized on the anion. These trends are clearly visualized in the charge density difference plots (Figure 2, right panels), which show pronounced electron accumulation on the anionic moiety upon adsorption and dissociation. The projected density of states, resolved for species, (Figure 2, central panels) further indicate that adsorption induces strong orbital delocalization compared to the isolated ion-pairs, with both ions contributing to low-energy unoccupied states. Consistently, the energy separation between the anion LUMO and the Fermi level of the Na(110) surface is smaller for FSI^–^ than TFSI^–^. Overall, such results rationalize the unique tendency of FSI^–^ anions toward reductive decomposition at Na surfaces.

To dissect the role of the cation on interfacial stability and reactivity, we also analyzed the corresponding Na-salts adsorption behavior at the Na (110) surface (Figure 3 and Figure S8 in SI). In this case, cation-Na^+^ and two side-on interaction modes are investigated for both NaFSI and NaTFSI salts. As for the IL, FSI-containing systems exhibit a pronounced tendency toward dissociation at the Na interface: FSI^–^ dissociation is observed for both *cis*-FSI and *trans*-FSI adsorption modes, while no dissociation occurs for NaTFSI in any configuration. Undissociated NaFSI adsorption energies range from −1.52 to −1.16 eV, while dissociated NaFSI configurations exhibit much more negative adsorption energies (−5.93 eV for *cis*-FSI and −4.98 to −6.47 eV for *trans*-FSI), which is also more exothermic than for PYR_13_FSI (Table S4 and Table S6 in SI). In contrast, NaTFSI shows significantly weaker adsorption, with *E*_ads_ values between −1.56 and −0.82 eV (*cis*) and-1.30 and −0.80 eV (*trans*). Structurally, the Na^+^ cation within the salt ion-pair interacts only weakly with the Na(110) surface, as evidenced by relatively long Na–Na distances (3.36–3.83 Å), whereas the anions chemisorb strongly via Na–O and Na–F bonds. For dissociated FSI^–^, the formation of short Na–F bonds (2.17–2.25 Å) highlights the strong affinity of fluoride species for the sodium surface, consistent with the IL adsorption results (see Table S7 in SI).

**Figure 3:**
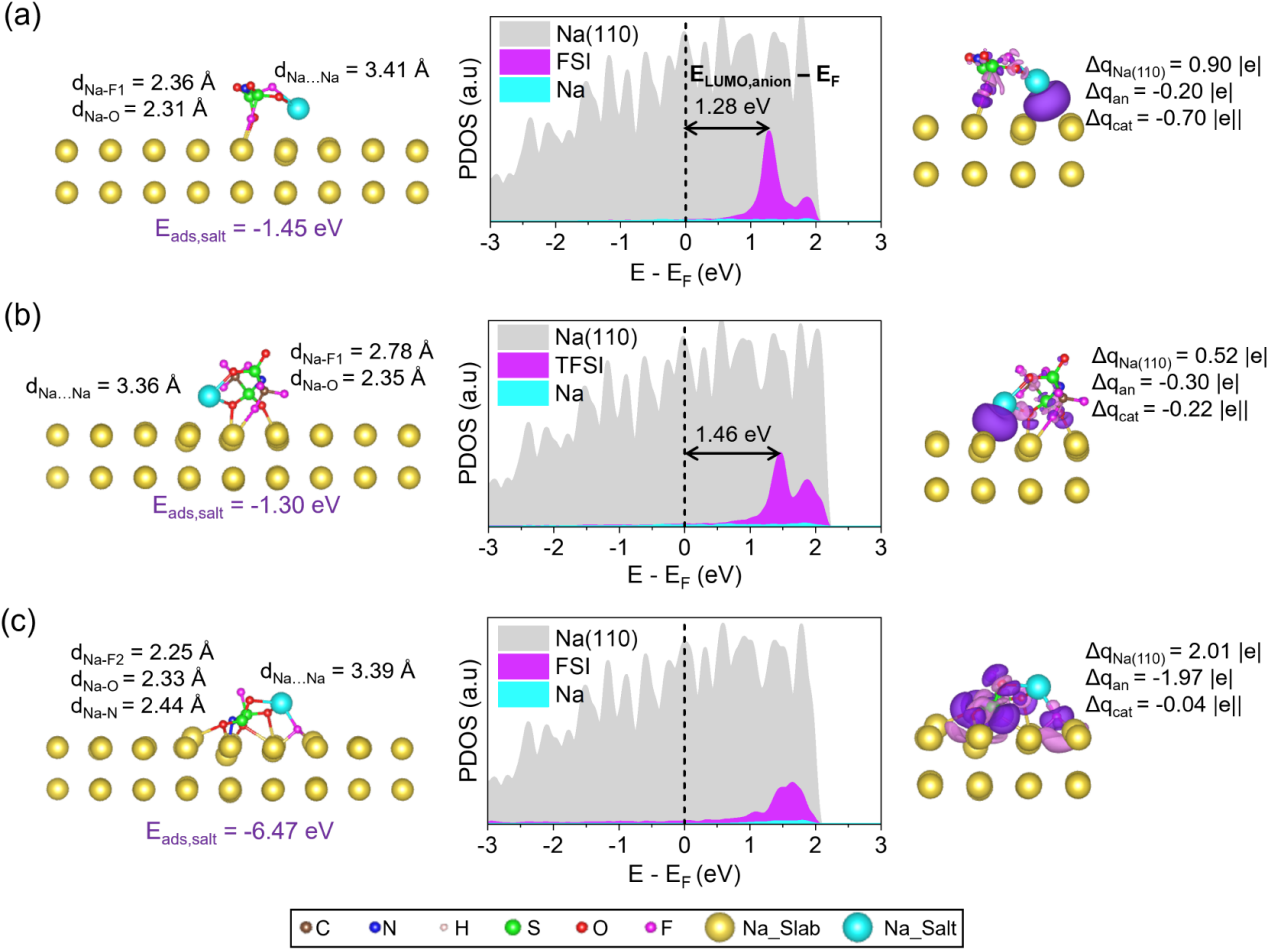
Optimized structures of the most stable adsorption configurations of the salts showing the minimum Na-slab/cation (and anion) distances and salt adsorption energies *E*_ads,salt_ (left), PDOS plots (middle), and CDD isosurfaces with the variations in the Bader charge-transfer values (right) of (a) NaFSI (*trans*-FSI) with undissociated FSI, (b) NaTFSI (*trans*-TFSI), and (c) NaFSI (*trans*-FSI) with dissociated FSI adsorbed on Na(110) surface. F1 and F2 represent the undissociated and dissociated F atoms, respectively. Atomic color code: C (brown), N (blue), H (white), S (green), O (red), F (magenta), Na within Na(110) slab (yellow), and Na within salt (cyan). CDD isosurface value: 0.0005 a.u, isosurface color code: positive (blue) and negative (pink). PDOS color codes: Na(110) slab (gray), Na within salt (cyan), and FSI/TFSI (purple). Theoretical level: PW/PBE.

Charge-transfer analysis reveals systematic electron transfer from the Na surface to the adsorbed salt species in all configurations (see Table S6 in SI). In contrast to the IL systems, where the PYR_13_^+^ cation typically acts as an electron donor, the salt adsorption results indicate that both Na^+^ and FSI^–^/TFSI^–^ act as electron acceptors relative to their isolated states, reflecting the metallic nature of the substrate and the strong polarization induced upon adsorption. Charge density difference plots (Figure 3, right panel) clearly visualize these effects, showing pronounced electron accumulation on the anionic moiety and depletion in the surface Na atoms, particularly in dissociated FSI^–^ configurations. Electronic structure analysis further supports these trends. PDOS plots (Figure 3, central panel) indicate significant adsorption-induced delocalization of frontier states, similar to the behavior observed for PYR_13_FSI/TFSI. However, in contrast to isolated salt ion-pairs, where the LUMO is largely cationic, the adsorbed systems show a dominant anionic contribution to the low-energy unoccupied states. Overall, such analysis further confirms the intrinsically higher reactivity of FSI^–^ compared to TFSI^–^ at sodium metal surfaces. Moreover, our results highlight that the FSI decomposition at Na interfaces can also be slightly modulated by the nature of the countercation, and it is enhanced by the presence of Na cations.

### 3.3 Structure and Dynamics of Electrolyte/Na(110) Interfaces

#### 3.3.1 FSI Decomposition from PYR_13_FSI and NaFSI

FSI^–^ in both PYR_13_FSI and NaFSI undergoes spontaneous dissociation for specific adsorption modes. To capture the finite-temperature interfacial dynamics, we performed AIMD simulations at 298 K starting from the most stable undissociated and dissociated FSI^–^ configurations identified earlier (Figures 2a and 2c for PYR_13_FSI, and Figures 3a and 3c for NaFSI). The complete AIMD trajectories (snapshots at 0.5 ps intervals) for systems starting from undissociated FSI^–^, with the corresponding temperature profiles and total energy fluctuation profiles, are shown in Figures S9 and S10. The temperature and energy fluctuation profiles confirm that the trajectories are well equilibrated and remain stable throughout the entire simulation period, while simultaneously capturing the dissociation dynamics at the electrolyte/Na(110) interface. In both systems, the temperature profiles show fluctuates strongly during the initial ∼0.75 ps and subsequently stabilize, indicating that the 5 ps simulation window adequately captures the equilibrated interfacial behavior.

For PYR_13_FSI (Figure S9), PYR_13_^+^ remains structurally intact and interacts weakly with both FSI^–^ and Na(110) surface throughout the simulation. Due to this weak interaction, PYR_13_ cation initially drifts away from the surface up to 5 Å at 1 ps, and it fluctuates within 2.55–3.83 Å, after equilibration. Meanwhile, FSI anion binds strongly with Na(110) surface, cordinating and extracting some surface Na atoms. Decomposition of FSI occurs around ∼0.75 ps, when both S–F bonds cleave nearly simultaneously. The system reaches an equilibrated N­(SO_2_)_2_ + 2F configuration by 1.5 ps. Subsequently, around 3.5 ps, one detached F atom migrates into the subsurface Na layers. No further decomposition is observed until 5 ps. The bond length evolution with time plot shown in Figure 4a (upper panel, left) further clarifies this process. Throughout the complete trajectory, the N–S bond lengths (initially ∼1.59 Å) remain almost unchanged, whereas the two S–F bonds (initially 1.61 and 1.68 Å) exhibit abrupt elongation to 5–6 Å near ∼0.75 ps, confirming F detachment. The corresponding radial distribution function (RDF) plot (Figure 4a, upper panel, middle) shows the interactions between Na atoms in the Na(110) surface and PYR_13_FSI molecule. Strong Na-FSI coordination follows the order Na–F > Na–O > Na–S > Na–N, while much weaker Na-PYR_13_ coordination is evident from the smaller Na–C and Na–H peaks. Charge-transfer evolution, evaluated at 1 ps intervals, further supports these trends, as shown in Figure 4a (upper panel, right). At 0 fs, Na(110), PYR_13_, and FSI exhibit charge-transfer values of 0.48, 0.68, and −1.16 |e| respectively (see Figure 2a, right panel). Upon FSI dissociation, substantial changes occur for Na(110) and FSI, after which the charges stabilize in the range from 3.68 to 3.96 |e| for Na(110) and −4.60 to −4.45 |e| for FSI. In contrast, PYR_13_ shows only minor variation (0.51–0.85 |e|), consistent with its weak interfacial interaction.

**Figure 4:**
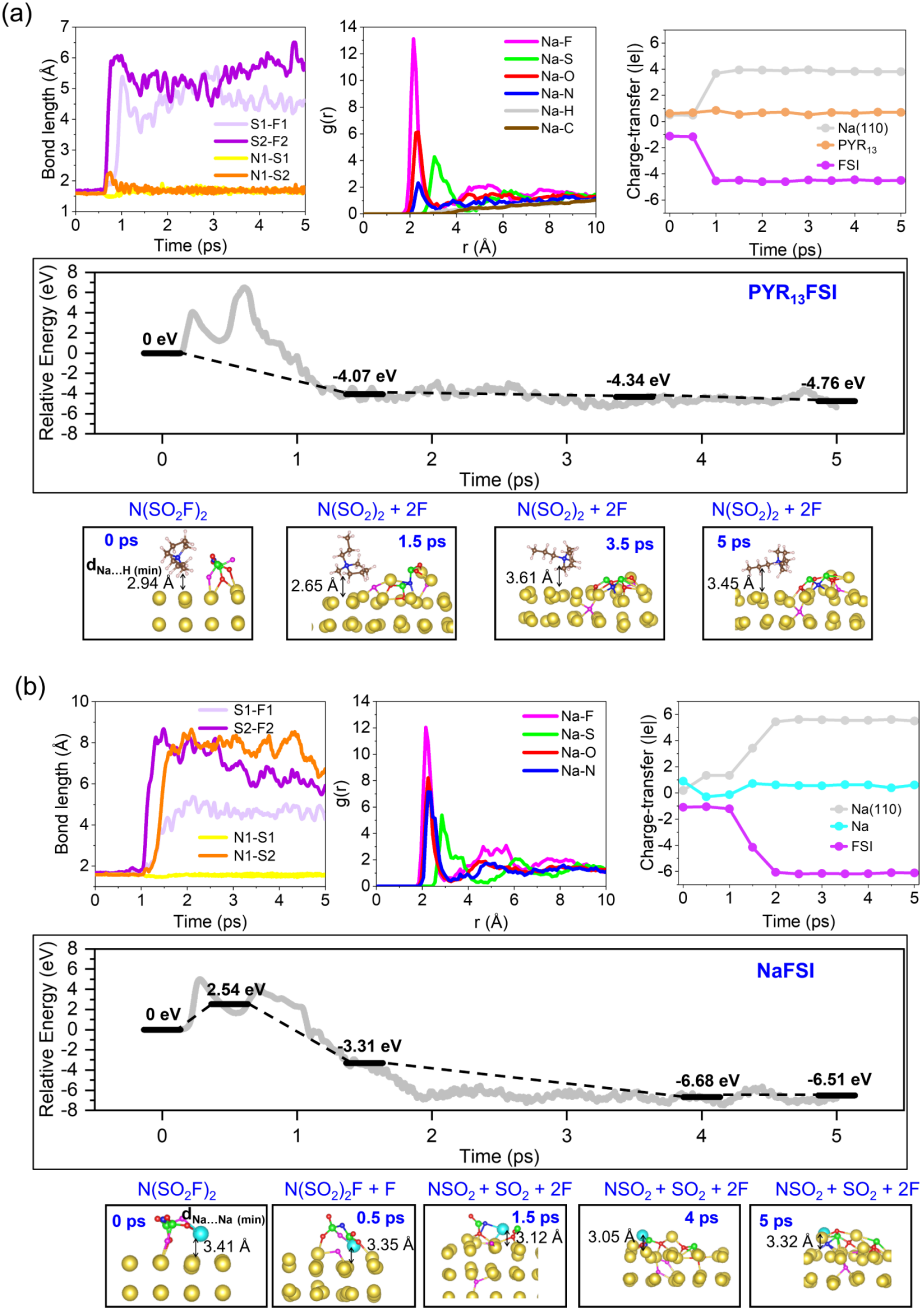
AIMD simulation illustrating the FSI decomposition pathway for (a) PYR_13_FSI with undissociated FSI initial state­(ref Figure 2a) *–* Upper panel: bond-length evolution with time (left), RDF plots showing coordination between Na atoms in Na(110) slab and atoms in PYR_13_ or decomposed FSI (middle), and Bader charge-transfer evolution with time (right); lower panel: Energetics and snapshots showing the primary decomposition steps with the distance between PYR_13_ and Na(110) surface (d_Na···H (min)_); Electronic energies extracted from AIMD trajectories are shown in gray color in the background, (b) NaFSI with undissociated FSI initial state­(ref Figure 3a) – Upper panel: bond-length evolution with time (left), RDF plots showing coordination between Na atoms in Na(110) slab and atoms in decomposed FSI (middle), and Bader charge-transfer evolution with time (right); lower panel: Energetics and snapshots showing the primary decomposition steps with distance between Na atom in salt and Na(110) surface (d_Na···Na (min)_); Electronic energies extracted from AIMD trajectories are shown in gray color in the background. Atomic color code: C (brown), N (blue), H (white), S (green), O (red), F (magenta), Na within Na(110) slab (yellow), and Na within salt (cyan).

The primary decomposed configurations extracted from the AIMD trajectory, along with their relative energies, are provided in Figure 4a (lower panel). Here, the relative energies are defined as the difference in the total energies of the corresponding configurations and that of the initial undissociated state at PBE-D3 level of theory at 0 K. The first fully dissociated configuration (two F atoms detached) exhibits a relative energy of −4.07 eV, indicating thermodynamically favorable spontaneous dissociation. Migration of one F atom into the Na(110) subsurface further stabilizes the system, reaching the final relative energy of −4.76 eV at 5 ps.

For NaFSI with initially undissociated FSI^–^, the complete AIMD trajectory (Figure S10) reveals behavior that differs from PYR_13_FSI. Throughout the simulation, the Na^+^ ion within NaFSI maintains a distance of 2.62–3.41 Å from Na(110) surface, whereas FSI interacts strongly with Na metal surface. The bond length evolution plot shown in Figure 4b (upper panel, left) reveals a stepwise FSI decomposition pathway. Slightly after 1 ps, the first S–F bond cleaves, increasing the bond distance from ∼1.6 Å to 8 Å, forming the first intermediate N­(SO_2_)_2_F + F configuration by 1.05 ps. Subsequently, between 1.2 and 1.5 ps, the second S–F bond and one N–S bond break nearly simultaneously, with bond lengths exceeding 8 Å and 4 Å, respectively. This leads to formation of the NSO_2_ + SO_2_ + 2F intermediate configuration by ∼1.5 ps, where one detached F atom migrates into the subsurface region of Na(110) slab. Following dissociation, one F atom migrates into the subsurface region of the Na(110) slab, and the second F atom also penetrates the slab, reaching an equilibrated configuration by ∼4 ps. Later, one F atom migrates back toward the top Na layer, and the second N–S bond remains almost constant throughout the simulation, indicating no further dissociation until 5 ps.

Consistent with these structural changes, the RDF plots in Figure 4b (upper panel, middle) infer strong Na-FSI coordination following the order Na-F > Na–O > Na–N > Na–S, accompanied by detachment of surface Na atoms. The charge-transfer profile in Figure 4b (upper panel, right) confirms the sequential decomposition steps. Initially at 0 ps, Na(110), Na^+^ (in salt), and FSI exhibit the charge-transfer values of 0.19, 0.90, and −1.09 |e|, respectively. Following the first F detachment (1.05 ps), these values change to 1.34 |e| for Na(110), −0.29 to −0.13 |e| for Na (in salt), and −1.21 to −1.05 |e| for FSI, highlighting slightly anionic nature of the Na atom in NaFSI. During the second decomposition step (1.2–1.5 ps), sharp transitions in the charge-transfer values occur, reaching 3.42, 0.72 and −4.15 |e|, respectively. After complete decomposition (beyond 1.5 ps), the charges stabilize in the range of 5.44–5.61 |e| (Na(110)), 0.39 to 0.64 |e| (Na (in salt)), and −6.18 to −6.06 |e| (FSI).

The relative energy profile shown in Figure 4b (lower panel) further confirms the thermodynamic evolution of the system. Interestingly, the first decomposition step exhibits a positive relative energy of 2.54 eV, indicating that the first F detachment from FSI is not intrinsically spontaneous. However, this positive energy is also attributed to intense surface reconstruction occurring at this early stage of simulation (∼1.05 ps). The next decomposition step forming NSO_2_ + SO_2_ + F lowers the relative energy to −3.31 eV, and the continued migration of F atoms into deeper Na layers stabilizes the system further to a minimum of −6.68 eV. The final geometry at 5 ps shows one F atom returning toward the top Na layer, with relative energy of −6.51 eV. Overall, the greater extent of fragmentation and higher mobility of F atoms in NaFSI indicate a more reactive interfacial behavior of FSI^–^ in the salt environment compared to the ionic liquid.

We next examine the FSI^–^ decomposition behavior for PYR_13_FSI and NaFSI starting from the partially dissociated FSI configuration, where one F atom is already detached and embedded within the Na layers at 0 ps (see Figures 2c and 3c). The complete AIMD trajectories along with the temperature and energy fluctuation profiles for these systems are provided in Figures S11 and S12, respectively. For PYR_13_FSI, the bond length evolution shown in Figure S13a (upper panel, left) indicates that the undissociated S–F bond (initially with bond length of 1.73 Å) dissociates ∼0.6 ps, evidenced by bond cleavage and a sudden increase in the S–F distance to ∼4.5 Å. In contrast, the other S–F and containing the already detached F atom (initially 3.33 Å) and the two N–S bonds (initially1.52 and 1.79 Å) remain nearly unchanged throughout the simulation, indicating no additional bond cleavage up to 5 ps. The fully decomposed configuration of N­(SO_2_)_2_ + 2F becomes equilibrated by ∼1.5 ps, with a reaction energy of −1.0 eV, as shown in Figure S13a (lower panel). The final structure at 5 ps exhibits a relative energy of 0.09 eV. Thus, regardless of whether FSI is initially intact or partially dissociated, the AIMD simulations converge to the same final product, N­(SO_2_)_2_ + 2F, within 5 ps. The corresponding RDF plots (Figure S13a, upper panel, middle) shows features similar to those observed for the undissociated starting geometry, indicating comparable coordination environments at the interface. Likewise, the charge-transfer evolution follows a similar trend (Figure S13a, upper panel, right). Initially, the Bader charge-transfer values at Na(110), PYR_13_, and FSI (with one F already detached) are 2.07, 0.80, and −2.87 |e|, respectively. Upon detachment of the second F atom (∼0.6 ps), the charge-transfer values for Na(110) and FSI shift abruptly and subsequently stabilize ranging from 3.71 to 4.01 |e| for Na(110), and −4.64 to −4.53 |e| for FSI. Charge-transfer for PYR_13_ remains nearly constant between 0.61 to 0.86 |e|.

To further verify the stability of N­(SO_2_)_2_ fragment, we extended the AIMD simulation for an additional 5 ps, using the fully dissociated N­(SO_2_)_2_ + 2F configuration as the starting geometry. No additional bond cleavage or structural transformation was observed up to 10 ps, confirming the intrinsic stability the N­(SO_2_)_2_ species. Representative snapshots at 5 ps (for both dissociated and undissociated initial conditions) and at 10 ps are provided in Table S8. Consistency in bond lengths and charge-transfer values across these structures further supports the identified decomposition pathway.

For NaFSI with partially dissociated FSI as the initial configuration, the behavior contrasts with that observed for the undissociated starting geometry discussed previously. In this case, the Na^+^ cation within NaFSI salt remains at a minimum distance of ranging 2.58–3.58 Å from Na(110) surface throughout the simulation. As shown in the bond length evolution in Figure S13b (upper panel, left), the N­(SO_2_)_2_F fragment remains intact for a longer period, persisting without additional decomposition up to ∼2.5 ps. The initial S–F bonds distances are 1.63 and 3.84 Å, and the two N–S bond lengths are 1.54 and 1.78 Å. As evident in Figure S13b, slightly after 2.5 ps the shorter S–F bond cleaves, with the bond distance increasing to ∼6.5 Å, whereas the longer S–F bond distance fluctuates within 3.5–5 Å. The two N–S bonds remain unchanged throughout, indicating no further decomposition.

The energetics plot (Figure S13b, lower panel) shows the resulting decomposed configuration reaches a of −0.19 eV by 3 ps. Subsequent structural reformation further stabilizes the system, lowering the relative energy to −1.53 eV by the end of 5 ps simulation. The RDF plot in Figure S13b (upper panel, middle) again indicates Na-FSI coordination in the order Na–F > Na–O > Na–S > Na–N, consistent with previous trends. The charge-transfer evolution plot in Figure S13b (upper panel, right) exhibits a similar trend. Initially, the charge-transfer values for Na(110), Na atom in NaFSI, and FSI are 2.02, 0.86, and −2.87 |e|, respectively. The Na^+^ charge slightly drops early in the simulation and remains stable within 0.82–0.90 |e| thereafter. On the contrary, until the initiation of the decomposition (0.5–2.5 ps), the charge-transfer values range as 2.02–2.13 |e| for Na(110) and −3.05 to −2.86 |e|. Afterward, the values change instantly at 2.5 ps and stabilize as 3.55–3.78 |e| for Na(110) and −4.58 to −4.45 |e| for FSI, upon S–F bond cleavage. Note that, unlike the NaFSI with undissociated starting geometry, we do not observe any N–S bond cleavage in this case.

#### 3.3.2 TFSI Decomposition from PYR_13_TFSI and NaTFSI

We now examine the behavior of TFSI^–^ on the Na(110) surface. As discussed previously, compared to FSI^–^, TFSI^–^ is more stable does not readily dissociate in either its *cis* or *trans* configurations across the adsorption modes of PYR_13_TFSI or NaTFSI (the most stable adsorption modes are previously shown in Figures 2b and 3b, respectively). This higher stability is consistent with earlier computational studies on TFSI-containing ILs on lithium surface.^29^ The complete AIMD trajectories, energy fluctuation and temperature profiles for PYR_13_TFSI and NaTFSI on Na(110) are provided in Figures S14 and S15, respectively.

As shown in the bond length evolution plot in Figure 5a (upper panel, left), PYR_13_ shows only weak interaction with the Na surface, drifting at distances of 2.59–4.49 Å, similar to the behavior observed for PYR_13_FSI. In contrast, TFSI interacts more strongly with the surface and undergoes delayed decomposition. The first decomposition occurs within ∼2–2.5 ps, producing NSO_2_CF_3_ and SO_2_CF_3_ fragments. This step is confirmed by the bond length evolution, where one of the two N–S bonds (initially 1.58 and 1.61 Å) cleaves at ∼2.5 ps, with the N–S distance increasing sharply to >5 Å. Notably, this first decomposed step is endothermic, with a positive relative energy of 1.36 eV, as shown in the energetics plot in Figure 5a (lower panel). To further investigate whether additional decomposition occurs, the AIMD simulation was extended by another 5 ps using the NSO_2_CF_3_ + SO_2_CF_3_ fragments as the starting point. Both fragments remain intact until the second decomposition step appears at 7.5–8 ps. At this stage, one of the two S–C bonds (initially 1.92 Å) breaks, increasing to ∼6 Å and releasing a CF_3_ moiety. The other S–C bond (initially 1.97 Å) remains intact. No further decomposition is observed up to 10 ps, marking the final decomposition product as NSO_2_CF_3_ + SO_2_ + CF_3_ visible by 8 ps with a relative energy of −0.48 eV. The larger bond-length fluctuations observed at approximately 9 ps in Figure 5a are associated with the rearrangement and surface migration of these dissociation fragments on Na(110), as illustrated by the representative snapshots reported in Figure S14, rather than with any further chemical decomposition. The final geometry at 10 ps exhibits relative energy of 0.76 eV. Overall, the delayed onset of decomposition and the positive configurational energy associated with the first dissociation step highlight the comparatively higher stability of TFSI^–^ on the Na(110) surface.

**Figure 5:**
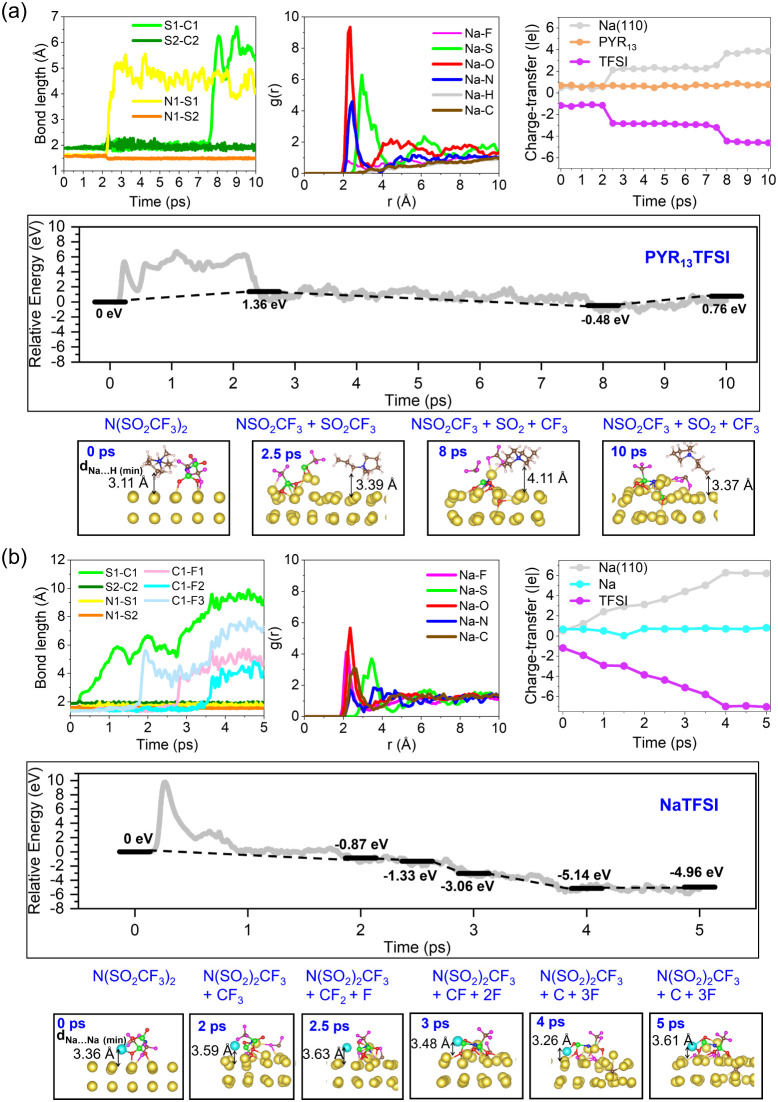
AIMD simulation illustrating the TFSI decomposition pathway for (a) PYR_13_TFSI (ref Figure 2b) *–* Upper panel: bond-length evolution with time (left), RDF plots showing coordination between Na atoms in Na(110) slab and atoms in PYR_13_ or decomposed TFSI (middle), and Bader charge-transfer evolution with time (right); lower panel: Energetics and snapshots showing the primary decomposition steps with the distance between PYR_13_ and Na(110) surface (d_Na···H (min)_); Electronic energies extracted from AIMD trajectories are shown in gray color in the background, (b) NaTFSI (ref Figure 3b) – Upper panel: bond-length evolution with time (left), RDF plots showing coordination between the Na atoms in Na(110) slab and atoms in decomposed TFSI (middle), and Bader charge-transfer evolution with time (right); Lower panel: Energetics and snapshots showing the primary decomposition steps with distance between Na atom in salt and Na(110) surface (d_Na···Na (min)_); Electronic energies extracted from AIMD trajectories are shown in gray color in the background. Atomic color code: C (brown), N (blue), H (white), S (green), O (red), F (magenta), Na within Na(110) slab (yellow), and Na within salt (cyan).

The RDF plots in Figure 5a (upper panel, middle) reveal the Na-TFSI coordination sequence as Na–O > Na–N > Na–S > Na–F > Na–C. The charge-transfer values of the starting geometry have been previously shown in Figure 2b as 0.48 |e| for Na(110), 0.69 |e| for PYR_13_, and −1.17 |e| for TFSI. As shown in Figure 5a (upper panel, right), PYR_13_ maintains an almost constant charge-transfer value (−0.48–0.72 |e|), while Na(110) and TFSI remain stable prior to decomposition (0.36–0.62 |e| and −1.25 to −1.09 |e|, respectively). At the moment of N–S bond cleavage, both values change significantly and later stabilize at 2.18–2.36 |e| for Na(110) and −2.86 to −2.81 |e| for TFSI. Bader charge-transfer values for Na(110) and TFSI^–^ remain stable between 5 and 7.5 ps (2.18–2.57 |e| and −2.96 to −2.84 |e|, respectively), shifting to 3.64–3.86 |e| and −4.63 to −4.46 |e| following S–C bond cleavage. As expected, PYR_13_ exhibits minimal change in charge-transfer throughout the extended trajectory, remaining between 0.52 and 0.77 |e| over the full 5–10 ps interval.

For NaTFSI, the Na^+^ ion remains positioned at 3.05–3.65 Å above the Na surface throughout the simulation, while TFSI^–^ undergoes a multistep decomposition process, as shown in Figure 5b. The decomposition initiates with the cleavage of one of the two S–C bonds (each initially 1.88 Å). As shown in the bond length evolution plot in Figure 5b (upper panel, left), this bond ruptures completely at ∼0.5–1 ps, increasing S–C distance to 4–6 Å and eventually ∼9 Å by 4 ps. In contrast, the second S–C bond remains intact, yielding N­(SO_2_)_2_CF_3_ and CF_3_ fragments as the first decomposition products, with relative energy of −0.87 eV (Figure S5b, lower panel). The subsequent decomposition step occurs within 1.5–2 ps (relative energy of −1.33 eV), where the F atoms begin to detach from the dissociated CF_3_ group. The initial C–F bond lengths are 1.34. 1.35, and 1.36 Å. The remaining two F atoms detach at 2.5–3 ps and 3.5–4 ps, with corresponding relative energies of −3.06 and −5.14 eV, respectively. This stepwise fragmentation ultimately produces N­(SO_2_)_2_CF_3_ + C + F + F + F as the final decomposition product. The final geometry at 5 ps exhibits a relative energy of −4.96 eV. Interestingly, unlike PYR_13_TFSI, where TFSI decomposition is delayed and partially endothermic, NaTFSI exhibits spontaneous and more extensive fragmentation of TFSI, highlighting the higher interfacial reactivity of the anion in salt environment.

The RDF plot in Figure 5b (upper panel, middle) indicates that the Na-TFSI coordination follows the sequence Na–O > Na–F > Na–C > Na–S > Na–N. The corresponding charge-transfer evolution

(Figure 5b, upper panel, right) further reflects the progressive decomposition process. Starting from the initial charge-transfer values of 0.52 |e| for Na(110) and −1.19 |e| for TFSI, the Na slab shows a gradual increase in charge accumulation, reaching 6.23 |e| by 5 ps, while TFSI decreases its charge −7.02 |e|. Meanwhile, the Na^+^ ion, starting at 0.67 |e|, maintains a nearly constant charge-transfer ranging 0.52–0.82 |e|. No further dissociation is observed upon extending the AIMD simulation until 10 ps, corroborating the stability of N­(SO_2_)_2_CF_3_ moiety. Snapshots of the trajectories at 5 and 10 ps are compared in Table S9. Overall, these results demonstrate that while TFSI^–^ is intrinsically more stable than FSI^–^, its decomposition on Na(110) becomes significantly more pronounced in the salt environment, emphasizing the crucial role of the ionic environment in governing the early interfacial reactions that contribute to SEI formation.

It should be noted that explicit bulk electrolyte effects were not included in the present simulations. Instead, the study focuses on the intrinsic reactivity of the electrolyte constituent molecules at the metal–electrolyte interface. The computational models were constructed with zero net charge, and the electrode potential was not explicitly controlled. As a result, the simulations represent interfacial behavior under conditions resembling open-circuit states. This approximation is particularly relevant because the initial stages of solid electrolyte interphase (SEI) formation are widely believed to occur under open-circuit or near-equilibrium conditions, prior to significant polarization of the electrode. Our finding highlighted the behavior as an electron donor of the Na metal surfaces. While the explicit inclusion of bulk solvent environments, electrolyte concentration effects, and applied electrode potentials could further refine the description of interfacial polarization and reaction energetics, addressing these aspects would require significantly larger-scale simulations and is beyond the scope of the present work, representing an important direction for future work. Nevertheless, the present results provide fundamental mechanistic insight into the stability and decomposition pathways of electrolyte constituent molecules at metal–electrolyte interfaces, which represent the primary elementary processes underlying the early stages of SEI formation.

## 4 Conclusions

In this work, we have systematically examined the initial stages of solid-electrolyte interphase (SEI) formation in ionic liquid (IL)-based electrolytes on a sodium metal anode using a combined density functional theory (DFT) and ab initio molecular dynamics (AIMD) approach. By explicitly considering the interactions and decomposition pathways of both IL solvent molecules and salt ion-pairs on the Na surface, this work provides a comprehensive atomistic picture of the earliest SEI-forming events. Two representative anions, FSI^–^ and TFSI^–^, paired with PYR_13_^+^ in the IL and Na^+^ in the salt, were investigated. Gas-phase calculations employing two different functionals show that although *trans* conformers of both anions are more stable than their *cis* analogues, no systematic trend emerges in their interaction strengths or HOMO–LUMO gaps. Consistent with previous studies, FSI^–^ and TFSI^–^ exhibit similar interactions with both PYR_13_^+^ and Na^+^.

Spin-polarized DFT calculations of various adsorption geometries reveal that side-on configurations are the most stable on the Na(110) surface. FSI^–^ exhibits pronounced reactivity in both PYR_13_FSI and NaFSI systems, including spontaneous F atom detachment during 0 K optimization, whereas TFSI^–^ remains intact in all adsorption modes. PYR_13_^+^ interacts only weakly with the Na surface, as confirmed by projected density of states (PDOS) and Bader charge analyses. No significant differences are observed between *cis* and *trans* conformers in adsorbed IL or salt ion-pairs.

To capture a realistic interfacial environment, AIMD simulations at 298 K were performed for both dissociated and intact anion configurations. In all cases, the PYR_13_^+^ and Na^+^ cations remain approximately 3–6 Å from the metal surface, indicating that the IL cation does not participate in the earliest stages of SEI formation. Owing to its higher reactivity, FSI^–^ decomposes rapidly (∼5–10 ps). For PYR_13_FSI, both intact and predissociated initial states evolve to the same final product, [N­(SO_2_)_2_ + 2F]. NaFSI yields the same product when starting from a dissociated configuration, whereas an undissociated initial geometry produces NSO_2_ + SO_2_ + 2F. In contrast, PYR_13_TFSI exhibits delayed decomposition, with the first event occurring at ∼1.5–2 ps (forming N­(SO_2_)_2_CF_3_ + SO_2_CF_3_) and a second at ∼7.5 ps (yielding NSO_2_CF_3_ + SO_2_ + CF_3_). The salt NaTFSI is more reactive, releasing a CF_3_ group within ∼1.5–2 ps to form N­(SO_2_)_2_CF_3_ + CF_3_, followed by CF_3_ fragmentation into three F atoms and one C atom by ∼4 ps. Across all IL and salt systems examined, NaF emerges as the dominant inorganic decomposition product.

Taken together, these results elucidate the fundamental anion-driven pathways governing early SEI formation in IL-based sodium electrolytes and provide valuable molecular-level guidelines for designing more stable and passivating electrolyte chemistries for next-generation Na-ion batteries. Moreover, the mechanistic insights obtained here also pave the way for future dynamic investigations employing larger and more realistic room-temperature ionic liquid (RTIL) models, enabling exploration of collective interfacial effects, long-time evolution, and mesoscale SEI development.

## Supplementary Material



## Notes

(i) surface energies and relative stabilities of Na surfaces; (ii) optimized structures, structural parameters, and charge-density difference plots of isolated ions and IL/salt ion pairs containing *cis* and *trans* FSI and TFSI anions; (iii) structural parameters and adsorption energies of various adsorption configurations of IL and salt ion-pairs on Na(110); (iv) complete AIMD (NVT) simulation data, including full trajectories, temperature-versus-time profiles, and total energy fluctuation-versus-time plots (PDF)

## References

[1] Duffner F., Kronemeyer N., Tübke J., Leker J., Winter M., Schmuch R. (2021). Post-Lithium-Ion Battery Cell Production and Its Compatibility
with Lithium-Ion Cell Production Infrastructure. Nat. Energy.

[2] Hwang J.-Y., Myung S.-T., Sun Y.-K. (2017). Sodium-Ion
Batteries: Present and
Future. Chem. Soc. Rev..

[3] Elbinger L., Enke M., Ziegenbalg N., Brendel J. C., Schubert U. S. (2024). Beyond
Lithium-Ion Batteries: Recent Developments in Polymer-Based Electrolytes
for Alternative Metal-Ion-Batteries. Energy
Storage Mater..

[4] Yao A., Benson S. M., Chueh W. C. (2025). Critically Assessing Sodium-Ion Technology
Roadmaps and Scenarios for Techno-Economic Competitiveness against
Lithium-Ion Batteries. Nat. Energy.

[5] Matei
Ghimbeu C., Beda A., Réty B., El Marouazi H., Vizintin A., Tratnik B., Simonin L., Michel J., Abou-Rjeily J., Dominko R. (2024). Review: Insights on
Hard Carbon Materials for Sodium-Ion Batteries (SIBs): Synthesis –
Properties – Performance Relationships. Adv. Energy Mater..

[6] Shen X., Liu H., Cheng X. B., Yan C., Huang J. Q. (2018). Beyond Lithium Ion
Batteries: Higher Energy Density Battery Systems Based on Lithium
Metal Anodes. Energy Storage Mater..

[7] Kang J. H., Lee J., Jung J. W., Park J., Jang T., Kim H. S., Nam J. S., Lim H., Yoon K. R., Ryu W. H., Kim I. D., Byon H. R. (2020). Lithium-Air
Batteries: Air-Breathing
Challenges and Perspective. ACS Nano.

[8] Mu X., He P., Zhou H. (2024). Toward Practical
Li-CO_2_ Batteries: Mechanisms,
Catalysts, and Perspectives. Acc. Mater. Res..

[9] Zhao M., Li B. Q., Zhang X. Q., Huang J. Q., Zhang Q. (2020). A Perspective
toward Practical Lithium-Sulfur Batteries. ACS
Cent. Sci..

[10] Sun J., O’Dell L. A., Armand M., Howlett P. C., Forsyth M. (2021). Anion-Derived
Solid-Electrolyte Interphase Enables Long Life Na-Ion Batteries Using
Superconcentrated Ionic Liquid Electrolytes. ACS Energy Lett..

[11] Zhao Y., Adair K. R., Sun X. (2018). Recent Developments
and Insights
into the Understanding of Na Metal Anodes for Na-Metal Batteries. Energy Environ. Sci..

[12] Zhao L., Costa S. I. R., Chen Y., Fitzpatrick J. R., Naylor A. J., Kolosov O., Tapia-Ruiz N. (2025). A Comparative
Study of Solid Electrolyte Interphase Evolution in Ether and Ester-Based
Electrolytes for Na -Ion Batteries. PRX Energy.

[13] Matios E., Wang H., Wang C., Li W. (2019). Enabling Safe Sodium
Metal Batteries by Solid Electrolyte Interphase Engineering: A Review. Ind. Eng. Chem. Res..

[14] Gao Y., Yao Y., Shi P., Huang F., Jiang Y., Yu Y. (2025). Advanced Interphases
Layers for Dendrite-Free Sodium Metal Anodes. ACS Appl. Mater. Interfaces.

[15] Eshetu G. G., Grugeon S., Laruelle S., Boyanov S., Lecocq A., Bertrand J. P., Marlair G. (2013). In-Depth Safety-Focused Analysis
of Solvents Used in Electrolytes for Large Scale Lithium Ion Batteries. Phys. Chem. Chem. Phys..

[16] Jamil R., Loomba S., Kar M., Collis G. E., Silvester D. S., Mahmood N. (2024). Metal Anodes Meet Ionic
Liquids: An Interfacial Perspective. Appl. Phys.
Rev..

[17] Stigliano P., Ferrara C., Pianta N., Gentile A., Mezzomo L., Lorenzi R., Berbenni V., Ruffo R., Appetecchi G. B., Mustarelli P. (2022). Physicochemical
Properties of Pyr13TFSI-NaTFSI Electrolyte
for Sodium Batteries. Electrochim. Acta.

[18] Hosokawa T., Matsumoto K., Nohira T., Hagiwara R., Fukunaga A., Sakai S., Nitta K. (2016). Stability of Ionic Liquids against
Sodium Metal: A Comparative Study of 1-Ethyl-3-Methylimidazolium Ionic
Liquids with Bis­(Fluorosulfonyl)­Amide and Bis­(Trifluoromethylsulfonyl)­Amide. J. Phys. Chem. C.

[19] Wang Y., Zaghib K., Guerfi A., Bazito F. F. C., Torresi R. M., Dahn J. R. (2007). Accelerating Rate
Calorimetry Studies of the Reactions
between Ionic Liquids and Charged Lithium Ion Battery Electrode Materials. Electrochim. Acta.

[20] Seh Z. W., Sun J., Sun Y., Cui Y. (2015). A Highly Reversible Room-Temperature
Sodium Metal Anode. ACS Cent. Sci..

[21] Shkrob I. A., Marin T. W., Zhu Y., Abraham D. P. (2014). Why Bis­(Fluorosulfonyl)­Imide
Is a “Magic Anion” for Electrochemistry. J. Phys. Chem. C.

[22] Rakov D. A., Chen F., Ferdousi S. A., Li H., Pathirana T., Simonov A. N., Howlett P. C., Atkin R., Forsyth M. (2020). Engineering
High-Energy-Density Sodium Battery Anodes for Improved Cycling with
Superconcentrated Ionic-Liquid Electrolytes. Nat. Mater..

[23] Ferdousi S. A., O’Dell L. A., Hilder M., Barlow A. J., Armand M., Forsyth M., Howlett P. C. (2021). SEI Formation on
Sodium Metal Electrodes
in Superconcentrated Ionic Liquid Electrolytes and the Effect of Additive
Water. ACS Appl. Mater. Interfaces.

[24] Javed F., Ullah F., Zakaria M. R., Akil H. M. (2018). An Approach to Classification
and Hi-Tech Applications of Room-Temperature Ionic Liquids (RTILs):
A Review. J. Mol. Liq..

[25] Fasulo F., Muñoz-García A. B., Massaro A., Crescenzi O., Huang C., Pavone M. (2023). Vinylene Carbonate
Reactivity at
Lithium Metal Surface: First-Principles Insights into the Early Steps
of SEI Formation. J. Mater. Chem. A.

[26] Massaro A., Avila J., Goloviznina K., Rivalta I., Gerbaldi C., Pavone M., Costa Gomes M. F., Padua A. A. H. (2020). Sodium Diffusion
in Ionic Liquid-Based Electrolytes for Na-Ion Batteries: The Effect
of Polarizable Force Fields. Phys. Chem. Chem.
Phys..

[27] Li Y., Leung K., Qi Y. (2016). Computational Exploration of the
Li-Electrode|Electrolyte Interface in the Presence of a Nanometer
Thick Solid-Electrolyte Interphase Layer. Acc.
Chem. Res..

[28] Zeng Y., Zhang S., Yao Y., Liu J., Zhou N., Tang J., Zhang K., Li Y., Hu J., Yang B., Peng C., Liu H. (2024). Structure and Interaction
of Ionic Liquid Monolayer on Lithium from First-Principles. J. Phys. Chem. C.

[29] Clarke-Hannaford J., Breedon M., Rüther T., Spencer M. J. S. (2020). Stability of
Boronium Cation-Based Ionic Liquid Electrolytes on the Li Metal Anode
Surface. ACS Appl. Energy Mater..

[30] Clarke-Hannaford J., Breedon M., Best A. S., Spencer M. J. S. (2019). The Interaction
of Ethylammonium Tetrafluoroborate [EtNH_3_
^+^]­[BF_4_
^-^] Ionic Liquid on the Li(001) Surface: Towards
Understanding Early SEI Formation on Li Metal. Phys. Chem. Chem. Phys..

[31] Lu Y., Tian Y., Xu Z., Liu H. (2022). Interfacial Structures
and Decomposition Reactions of Hybrid Anion-Based Ionic Liquids at
Lithium Metal Surface from First-Principles and Ab Initio Molecular
Dynamics. J. Mol. Liq..

[32] Yildirim H., Haskins J. B., Bauschlicher C. W., Lawson J. W. (2017). Decomposition of
Ionic Liquids at Lithium Interfaces. 1. Ab Initio Molecular Dynamics
Simulations. J. Phys. Chem. C.

[33] Haskins J. B., Yildirim H., Bauschlicher C. W., Lawson J. W. (2017). Decomposition of
Ionic Liquids at Lithium Interfaces. 2. Gas Phase Computations. J. Phys. Chem. C.

[34] Merinov B. V., Zybin S. V., Naserifar S., Morozov S., Oppenheim J., Goddard W. A., Lee J., Lee J. H., Han H. E., Choi Y. C., Kim S. H. (2019). Interface
Structure in Li-Metal/[Pyr_14_]­[TFSI]-Ionic Liquid System
from Ab Initio Molecular Dynamics
Simulations. J. Phys. Chem. Lett..

[35] Ayatollahi S. F., Bahrami M., Ghatee M. H., Ghaed-Sharaf T. (2020). Simulating
the Effect of Anions on Spreading of Nanodroplets and the Monolayer
Behavior of Quaternary Ammonium-Based Ionic Liquids on Li(100) and
Li(110) Metal Facets. Ind. Eng. Chem. Res..

[36] Angarita-Gomez S., Balbuena P. B. (2022). Lithium-Ion Transport through Complex Interphases in
Lithium Metal Batteries. ACS Appl. Mater. Interfaces.

[37] Perdew J. P., Burke K., Ernzerhof M. (1997). Generalized Gradient Approximation
Made Simple. Phys. Rev. Lett..

[38] Becke A. D. (1993). Density-
Functional Thermochemistry III. The Role of Exact Exchange. J. Chem. Phys..

[39] Kresse G., Furthmüller J. (1996). Efficiency
of Ab-Initio Total Energy Calculations for
Metals and Semiconductors Using a Plane-Wave Basis Set. Comput. Mater. Sci..

[40] Kresse G., Hafner J. (1994). Ab Initio Molecular-Dynamics
Simulation of the Liquid-Metal-Amorphous-Semiconductor
Transition in Germanium. Phys. Rev. B.

[41] Kresse G., Hafner J. (1993). Ab *initio* Molecular Dynamics for Liquid
Metals. Phys. Rev. B.

[42] Kresse G., Furthmüller J. (1996). Efficient Iterative Schemes for Ab Initio Total-Energy
Calculations Using a Plane-Wave Basis Set. Phys.
Rev. B.

[43] Bengtsson L. (1999). Dipole Correction
for Surface Supercell Calculations. Phys. Rev.
B.

[44] Gaissmaier D., van den Borg M., Fantauzzi D., Jacob T. (2020). Microscopic Properties
of Na and Li—A First Principle Study of Metal Battery Anode
Materials. ChemSusChem.

[45] Henkelman G., Arnaldsson A., Jónsson H. (2006). A Fast and Robust Algorithm for Bader
Decomposition of Charge Density. Comput. Mater.
Sci..

[46] Momma K., Izumi F. (2011). VESTA 3 for Three-Dimensional
Visualization of Crystal, Volumetric
and Morphology Data. J. Appl. Crystallogr..

[47] Humphrey W., Dalke A., Schulten K. (1996). VMD: Visual Molecular Dynamics. J. Mol. Graph..

[48] Johansson P., Fast L. E., Matic A., Appetecchi G. B., Passerini S. (2010). The Conductivity of Pyrrolidinium
and Sulfonylimide-Based
Ionic Liquids: A Combined Experimental and Computational Study. J. Power Sources.

[49] Kufel L., Cid R., Bonilla F. J., Chen F., Somers A. E., Forsyth M., Pringle J. M. (2025). Modulation
of Sodium-Metal Interphase through Ionic
Liquid Electrolyte Chemistry and Formation Current. ACS Appl. Energy Mater..

[50] Arı H., Büyükmumcu Z. (2017). Comparison
of DFT Functionals for
Prediction of Band Gap of Conjugated Polymers and Effect of HF Exchange
Term Percentage and Basis Set on the Performance. Comput. Mater. Sci..

[51] Zhang G., Musgrave C. B. (2007). Comparison of DFT
Methods for Molecular Orbital Eigenvalue
Calculations. J. Phys. Chem. A.

[52] Gao W., Abtew T. A., Cai T., Sun Y. Y., Zhang S., Zhang P. (2016). On the Applicability
of Hybrid Functionals for Predicting Fundamental
Properties of Metals. Solid State Commun..

