# Supplementary material for: Adsorption and Reactivity of Na+ Salt and Room-Temperature Ionic Liquid at Na Metal Surface
Source: J Phys Chem C Nanomater Interfaces. 2026 Jul 19;130(30):10520–32. doi: 10.1021/acs.jpcc.6c02391 (PMC13435593; doi:10.1021/acs.jpcc.6c02391)
Supplement: Supplementary file 1 [file jp6c02391_si_001.pdf]

Supplementary Information

Adsorption and Reactivity of Na^+ Salt and Room-Temperature Ionic Liquid at Na Metal Surface

Ranjini Sarkar,[1] Francesca Fasulo,[1,*] Ana Belén Muñoz-García,[1] Bernardino Tirri,[2] Alfonso Amendola,[2] Giacomo Melani,[3] Simone Piccinin,[4] and Michele Pavone.[5,*]

[1]Department of Physics "E. Pancini", University of Naples Federico II, Comp. Univ. Monte Sant'Angelo Via Cintia 21, 80126 Napoli, Italy

[2]High Performance Computing Center of Excellence, Eni S.p.A., DIT - Digital & Information Technology, Via Felice Maritano, 26, 20097 San Donato Milanese (MI), Italy.

[3]New Energies, Renewable Energies and Materials Science Research Center, Instituto Guido Donegani, Eni S.p.A via Fauser 4, Novara I-28100, Italy.

[4]CNR - Istituto Officina dei Materiali, c/o SISSA, Via Bonomea 265, 34136 Trieste, Italy

[5]Department of Chemical Sciences, University of Naples Federico II, University of Naples Federico II, Comp. Univ. Monte Sant'Angelo Via Cintia 21, 80126 Napoli, Italy

* Corresponding authors: F.F. *francesca.fasulo@unina.it*; M.P. *michele.pavone@unina.it*

1. Surfaces stability of Na metal anode

Na slabs with lattice planes [100], [110], and [111] were cleaved from optimized Na bulk unit cell (lattice constant a = 4.17 Å), as shown in Figure S1. The surface energies (γ) of these facets were evaluated as a function of slab thickness, as:

$$\gamma = \frac{E_{slab} - nE_{bulk}}{2A}$$

where E_{slab}, E_{bulk}, n, and A are the total energy of the Na slab, energy of Na bulk per formula units, number of Na atoms in the slab, and surface area, respectively. Convergence of the surface energy with respect to slab thickness was achieved for a four-layer slab, which was therefore adopted for all subsequent calculations. Among the three lattice planes, we found that Na [110] is the most stable (Table S1), as previously reported. [1]

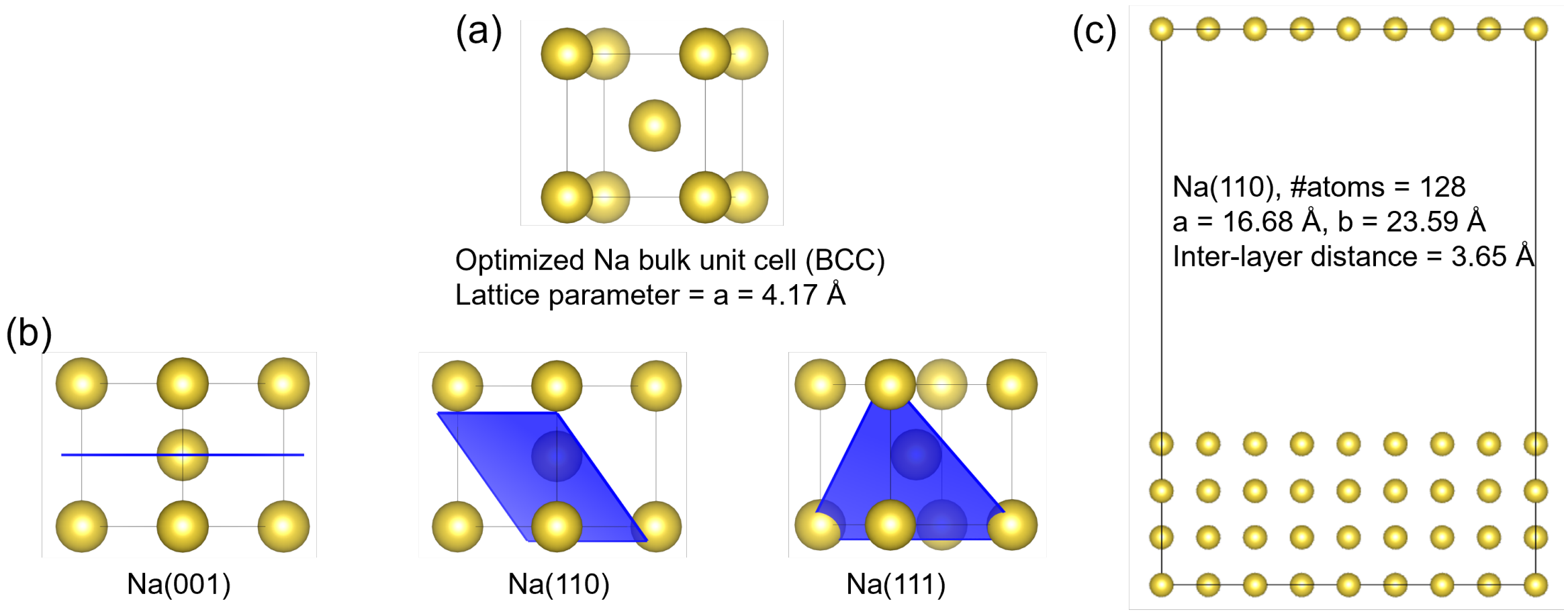

Figure S1. (a) Optimized structure of Na bulk unit cell, (b) Na(001), Na(110), and Na(111), (c) optimized structure of four-layered 4x4 Na(110) surface.

Table S1. Comparison of the surface energies (γ) of Na(100), (110), and (111) slabs as a function of slab thickness.

#layers	(100)		(110)		(111)	
	#atoms	γ (J/m^2)	#atoms	γ (J/m^2)	#atoms	γ (J/m^2)
2L	2	0.308	4	0.294	4	0.314
4L	4	0.310	8	0.306	8	0.342
5L	5	0.310	10	0.303	10	0.345
6L	6	0.307	12	0.303	12	0.352
8L	8	0.307	16	0.299	16	0.345

2. Geometry optimization of isolated anions (FSI^-, and $TFSI^-$) and cation (PYR_{13}^+)

Before constructing the ion-pair structures, we optimized the geometries of the individual charged species (FSI^-, $TFSI^-$, and PYR_{13}^+) comparing the results of the two different functionals: PBE and B3LYP with dispersion correction (DFT-D3). The optimized structures are showed in Figure S2, and the corresponding structural parameters are summarized in Table S2. Both cis and trans conformers of FSI^- and $TFSI^-$ anions are considered, following the approach by Zeng et al.[2] While bond lengths and bond angles remain essentially unchanged between the two conformations, the dihedral angles F–S–S–F (for FSI^-) and C–S–S–C (for $TFSI^-$) differ markedly. For FSI^-, the dihedral angles are 0.07° (cis) and 143° (trans) at the PBE level (0.06° and 144° at B3LYP), whereas for $TFSI^-$ they are 44° (cis) and 173° (trans) at PBE (44° and 172° at B3LYP). Overall, the structural parameters of all isolated ions obtained using the two computational approaches are highly consistent (Table S2).

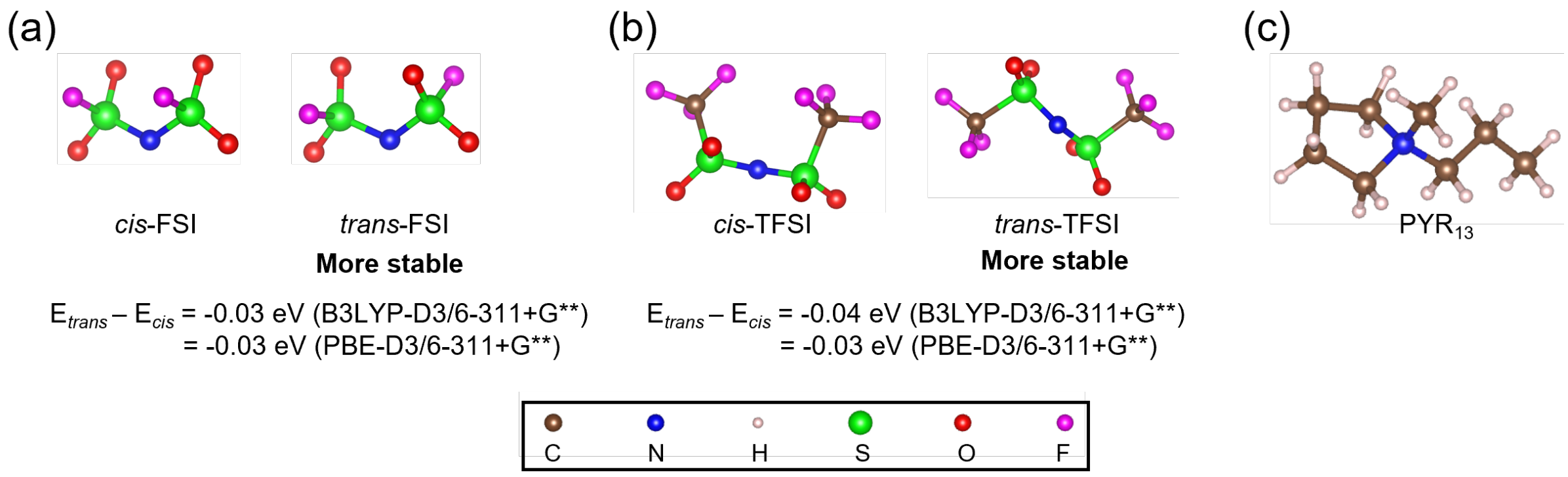

Figure S2. Structural representation of isolated (a) FSI, (b) TFSI, and (c) PYR_{13}. E_{trans} and E_{cis} denote the energies of the optimized geometries of isolated *trans* and *cis* configurations of anions, respectively. Atomic color code: C (brown), N (blue), H (white), S (green), O (red), and F (magenta).

Table S2. Structural parameters, i.e., bond lengths (d), bond angles (A), and dihedral angles (D) of isolated FSI^-, $TFSI^-$, and PYR_{13}^+ optimized using PBE and B3LYP functionals

**Systems**	**Structural Parameters**		**Systems**	**Structural Parameters**	
	PBE-D3	***B3LYP-D3***		***PBE-D3***	***B3LYP-D3***
cis-FSI	d_{N-S} = 1.62 Å	d_{N-S} = 1.60 Å	*trans*-FSI	d_{N-S} = 1.62 Å	d_{N-S} = 1.61 Å
	d_{S-F} = 1.70 Å	d_{S-F} = 1.67 Å		d_{S-F} = 1.70 Å	d_{S-F} = 1.67 Å
	d_{S-O} = 1.47 Å	d_{S-O} = 1.45 Å		d_{S-O} = 1.47 Å	d_{S-O} = 1.45 Å
	A_{S-N-S} = 123°	A_{S-N-S} = 124°		A_{S-N-S} = 122°	A_{S-N-S} = 123°
	$D_{F-S-S-F}$ = 0.07°	$D_{F-S-S-F}$ = 0.06°		$D_{F-S-S-F}$ = 143°	$D_{F-S-S-F}$ = 144°
cis-TFSI	d_{N-S} = 1.63 Å	d_{N-S} = 1.63 Å	*trans*-TFSI	d_{N-S} = 1.63 Å	d_{N-S} = 1.63 Å
	d_{S-C} = 1.91 Å	d_{S-C} = 1.90 Å		d_{S-C} = 1.91 Å	d_{S-C} = 1.90 Å
	d_{S-O} = 1.48 Å	d_{S-O} = 1.46 Å		d_{S-O} = 1.48 Å	d_{S-O} = 1.46 Å
	d_{C-F} = 1.35 Å	d_{C-F} = 1.34 Å		d_{C-F} = 1.35 Å	d_{C-F} = 1.34 Å
	A_{S-N-S} = 125°	A_{S-N-S} = 126°		A_{S-N-S} = 124°	A_{S-N-S} = 125°
	$D_{C-S-S-C}$ = 44°	$D_{C-S-S-C}$ = 44°		$D_{C-S-S-C}$ = 173°	$D_{C-S-S-C}$ = 172°
PYR_{13}	d_{C-H} = 1.10 Å	d_{C-H} = 1.09 Å			
	d_{N-C} = 1.53 Å	d_{N-C} = 1.52 Å			
	d_{C-C} = 1.54 Å	d_{C-C} = 1.53 Å			

3. Geometry optimization of isolated ILs and salts conformers

3a. IL and salt ion-pairs with *cis*-FSI and TFSI

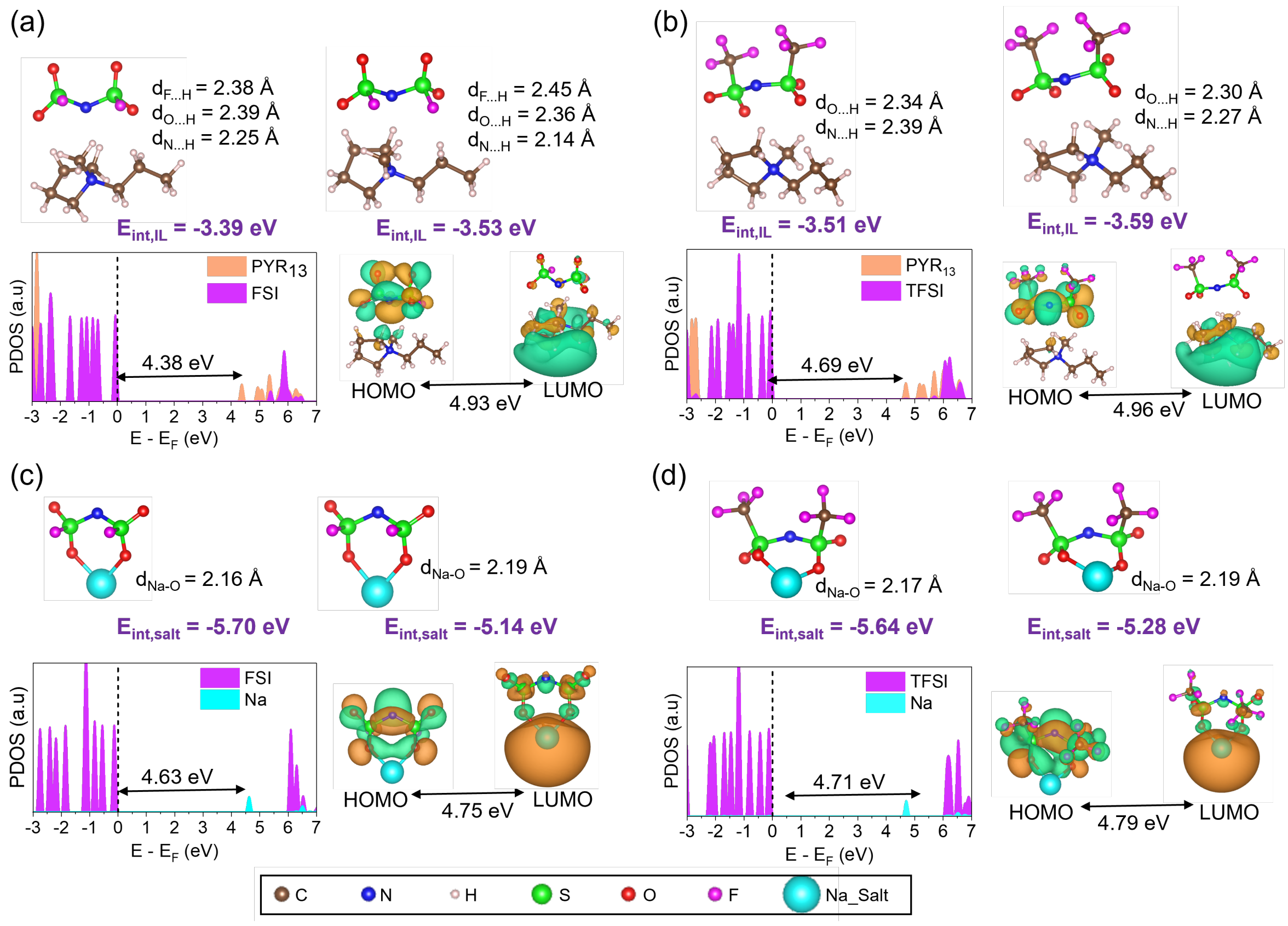

Figure S3. Minimum-energy structures and electronic structure of (a) $PYR_{13}FSI$ (*cis*-FSI), (b) $PYR_{13}TFSI$ (*cis*-TFSI), (c) NaFSI (*cis*-FSI) and (d) NaTFSI (*cis*-TFSI). For each panel, the left part is at the PW/PBE level of theory and right part at the GTO/PBE level. The minimum inter-ionic bond lengths, anion/cation interaction energies ($E_{int,IL}$ and $E_{int,salt}$), VBM-CBM gap, and HOMO-LUMO gaps for all the ion-pairs are reported. Atomic color code: C (brown), N (blue), H (white), S (green), O (red), F (magenta), Na within salt (cyan). HOMO-LUMO isosurface value: 0.02 a.u, isosurface color code: positive (orange) and negative (green). PDOS color codes: PYR_{13} (orange), Na within salt (cyan), and FSI/TFSI (purple).

3b. Geometry optimization of salt and ILs conformers with FSI and TFSI under B3LYP level of theory

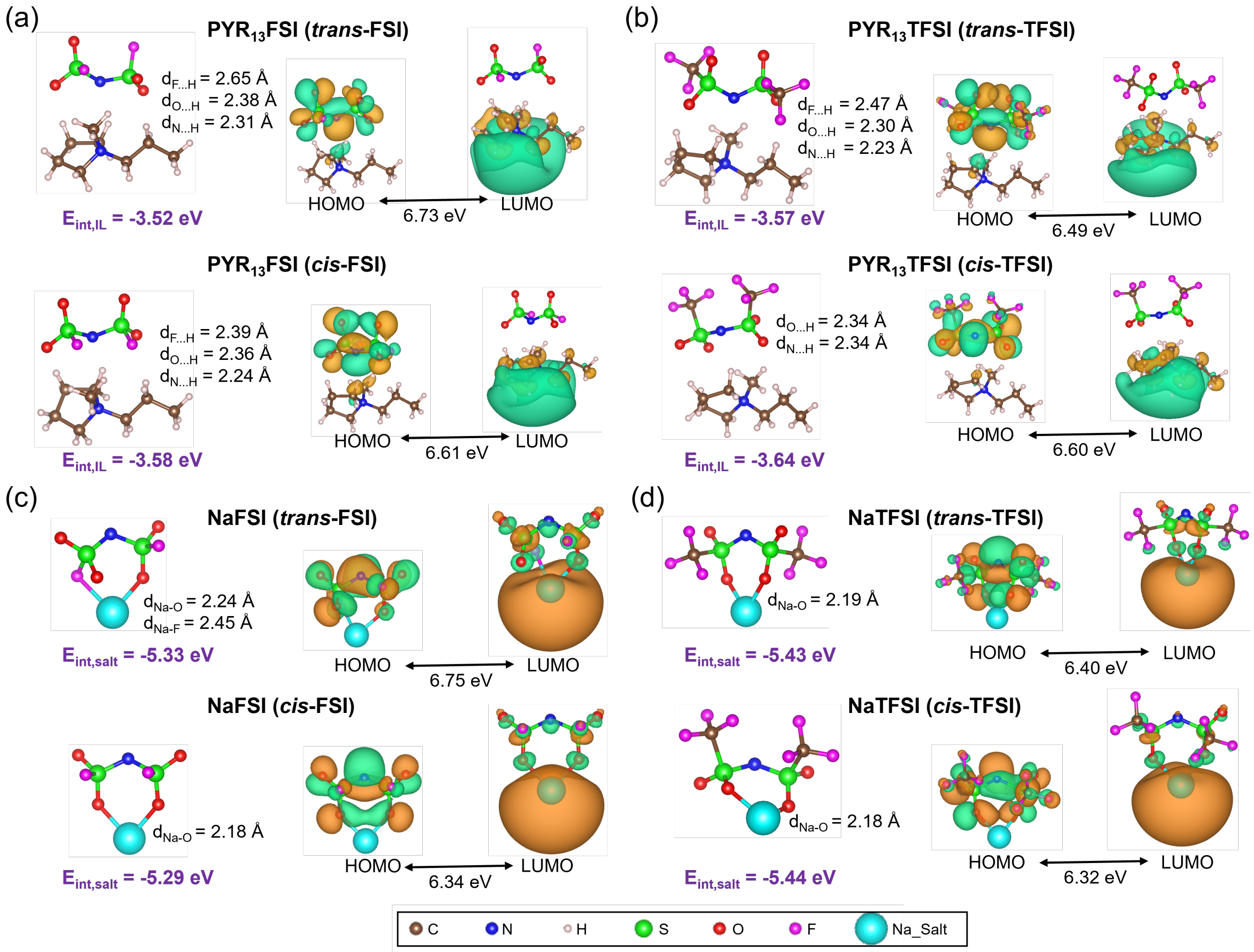

Figure S4. Optimized structures (left) and HOMO-LUMO plots (right) obtained at the GTO/B3LYP level of theory in the gas phase for both *cis* and *trans* anion-configurations of (a) $PYR_{13}FSI$, (b) $PYR_{13}FSI$, (c) NaFSI, and (d) NaTFSI. Atomic color code: C (brown), N (blue), H (white), S (green), O (red), F (magenta), Na within salt (cyan). HOMO-LUMO isosurface value: 0.02 a.u, isosurface color code: positive (orange) and negative (green).

3c. Charge-density-difference (CDD) analyses of IL and salt ion pairs

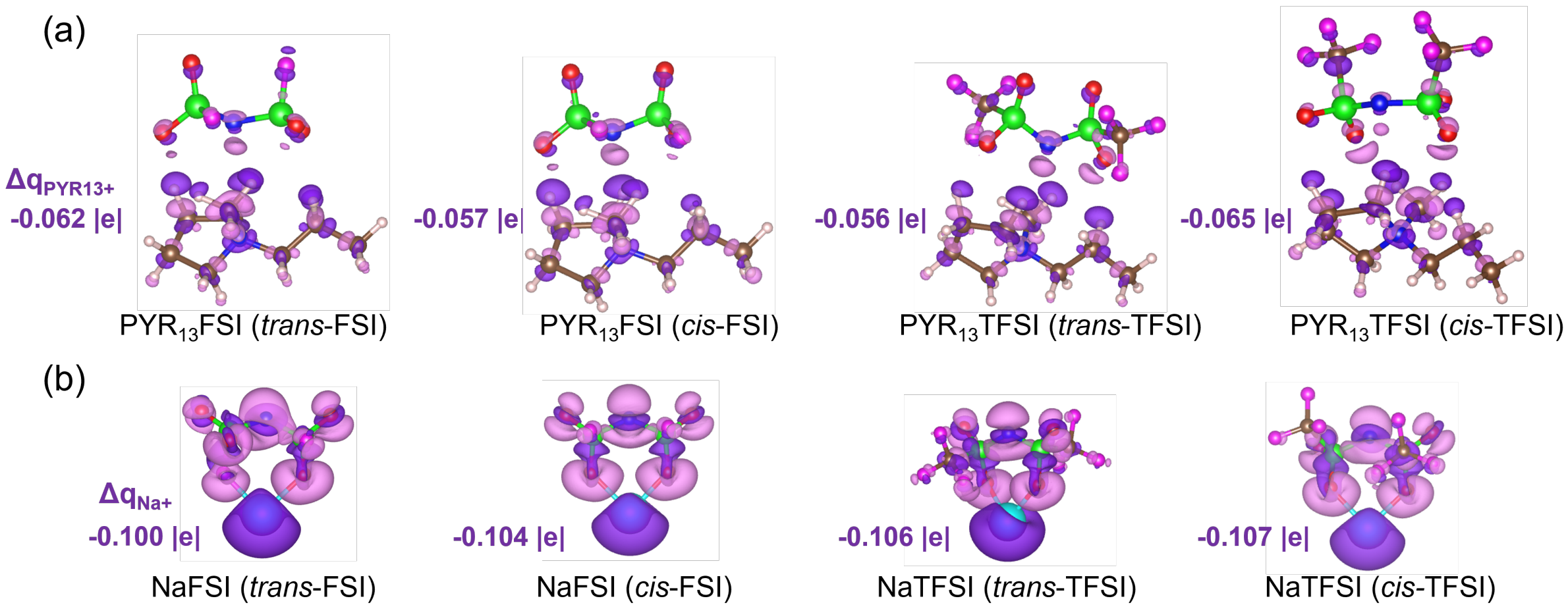

Figure S5. CDD plots of the isolated molecules of (a) ILs and (b) salts with the variations in Bader charge-transfers at the cations PYR_{13}^{+} and Na^{+} (denoted as Δq_{Na+} and ΔPYR_{13+}). Atomic color code: C (brown), N (blue), H (white), S (green), O (red), F (magenta), Na within salt (cyan). CDD isosurface value: 0.0005 a.u, isosurface color code: positive (blue) and negative (pink).

Table S3. Structural parameters, i.e., bond lengths (d), bond angles (A), and dihedral angles (D) of the FSI^-, $TFSI^-$, and PYR_{13}^+ within corresponding IL and salt ion-pairs optimized using different levels of theory

Ions	Structural Parameters					
	**PBE-D3/ 6-311+G****	**B3LYP-D3/ 6-311+G****	**GGA-PBE/ PAW**	**PBE-D3/ 6-311+G****	**B3LYP-D3/ 6-311+G****	**GGA-PBE/ PAW**
	IL ($PYR_{13}FSI$)			***Salt (NaFSI)***		
cis- FSI	d_{N-S} = 1.63 Å	d_{N-S} = 1.61 Å	d_{N-S} = 1.59 Å	d_{N-S} = 1.61 Å	d_{N-S} = 1.60 Å	d_{N-S} = 1.58 Å
	d_{S-F} = 1.70 Å	d_{S-F} = 1.67 Å	d_{S-F} = 1.64 Å	d_{S-F} = 1.67 Å	d_{S-F} = 1.64 Å	d_{S-F} = 1.62 Å
	d_{S-O} = 1.46 Å	d_{S-O} = 1.45 Å	d_{S-O} = 1.43 Å	d_{S-O} = 1.45 Å	d_{S-O} = 1.45 Å	d_{S-O} = 1.42 Å
	A_{S-N-S} = 124°	A_{S-N-S} = 125°	A_{S-N-S} = 126°	A_{S-N-S} = 124°	A_{S-N-S} = 125°	A_{S-N-S} = 126°
	D_{FSSF} = 12.7°	D_{FSSF} = 10.6°	D_{FSSF} = 11.6°	D_{FSSF} = 4.0°	D_{FSSF} = 3.4°	D_{FSSF} = 3.6°
PYR_{13}	d_{C-H} = 1.10	d_{C-H} = 1.09	d_{C-H} = 1.10	-	-	-
	d_{N-C} = 1.51	d_{N-C} = 1.52	d_{N-C} = 1.52			
	d_{C-C} = 1.53	d_{C-C} = 1.53	d_{C-C} = 1.53			
trans- FSI	d_{N-S} = 1.58 Å	d_{N-S} = 1.61 Å	d_{N-S} = 1.58 Å	d_{N-S} = 1.61 Å	d_{N-S} = 1.59 Å	d_{N-S} = 1.58 Å
	d_{S-F} = 1.60 Å	d_{S-F} = 1.64 Å	d_{S-F} = 1.61 Å	d_{S-F} = 1.66 Å	d_{S-F} = 1.64 Å	d_{S-F} = 1.61 Å
	d_{S-O} = 1.43 Å	d_{S-O} = 1.44 Å	d_{S-O} = 1.43 Å	d_{S-O} = 1.45 Å	d_{S-O} = 1.43 Å	d_{S-O} = 1.42 Å
	A_{S-N-S} = 127°	A_{S-N-S} = 125°	A_{S-N-S} = 127°	A_{S-N-S} = 119°	A_{S-N-S} = 121°	A_{S-N-S} = 125°
	D_{FSSF} = 103°	D_{FSSF} = 147°	D_{FSSF} = 104°	D_{FSSF} = 144°	D_{FSSF} = 145°	D_{FSSF} = 110°
PYR_{13}	d_{C-H} = 1.10 Å	d_{C-H} = 1.09 Å	d_{C-H} = 1.10 Å	-	-	-
	d_{N-C} = 1.51 Å	d_{N-C} = 1.51 Å	d_{N-C} = 1.51 Å			
	d_{C-C} = 1.54 Å	d_{C-C} = 1.53 Å	d_{C-C} = 1.53 Å			
	IL ($PYR_{13}TFSI$)			***Salt (NaTFSI)***		
cis- TFSI	d_{N-S} = 1.63 Å	d_{N-S} = 1.61 Å	d_{N-S} = 1.59 Å	d_{N-S} = 1.61 Å	d_{N-S} = 1.59 Å	d_{N-S} = 1.58 Å
	d_{S-C} = 1.91 Å	d_{S-C} = 1.89 Å	d_{S-C} = 1.88 Å	d_{S-C} = 1.91 Å	d_{S-C} = 1.90 Å	d_{S-C} = 1.88 Å
	d_{S-O} = 1.48 Å	d_{S-O} = 1.46 Å	d_{S-O} = 1.44 Å	d_{S-O} = 1.48 Å	d_{S-O} = 1.45 Å	d_{S-O} = 1.43 Å
	d_{C-F} = 1.35 Å	d_{C-F} = 1.35 Å	d_{C-F} = 1.34 Å	d_{C-F} = 1.35 Å	d_{C-F} = 1.33 Å	d_{C-F} = 1.35 Å
	A_{S-N-S} = 125°	A_{S-N-S} = 129°	A_{S-N-S} = 127°	A_{S-N-S} = 124°	A_{S-N-S} = 128°	A_{S-N-S} = 129°
	D_{CSSC} = 42°	D_{CSSC} = 173°	D_{CSSC} = 42°	D_{CSSC} = 170°	D_{CSSC} = 36°	D_{CSSC} = 37°
PYR_{13}	d_{C-H} = 1.10 Å	d_{C-H} = 1.09 Å	d_{C-H} = 1.09 Å	-	-	-
	d_{N-C} = 1.52 Å	d_{N-C} = 1.51 Å	d_{N-C} = 1.51 Å			
	d_{C-C} = 1.53 Å	d_{C-C} = 1.53 Å	d_{C-C} = 1.53 Å			
trans- TFSI	d_{N-S} = 1.64 Å	d_{N-S} = 1.62 Å	d_{N-S} = 1.60 Å	d_{N-S} = 1.62 Å	d_{N-S} = 1.60 Å	d_{N-S} = 1.58 Å
	d_{S-C} = 1.91 Å	d_{S-C} = 1.89 Å	d_{S-C} = 1.89 Å	d_{S-C} = 1.91 Å	d_{S-C} = 1.89 Å	d_{S-C} = 1.88 Å
	d_{S-O} = 1.48 Å	d_{S-O} = 1.45 Å	d_{S-O} = 1.44 Å	d_{S-O} = 1.48 Å	d_{S-O} = 1.45 Å	d_{S-O} = 1.48 Å
	d_{C-F} = 1.35 Å	d_{C-F} = 1.34 Å	d_{C-F} = 1.35 Å	d_{C-F} = 1.34 Å	d_{C-F} = 1.34 Å	d_{C-F} = 1.35 Å
	A_{S-N-S} = 124°	A_{S-N-S} = 125°	A_{S-N-S} = 126°	A_{S-N-S} = 126°	A_{S-N-S} = 127°	A_{S-N-S} = 128°
	D_{CSSC} = 170°	D_{CSSC} = 171°	D_{CSSC} = 172°	D_{CSSC} = 172°	D_{CSSC} = 166°	D_{CSSC} = 171°
PYR_{13}	d_{C-H} = 1.10 Å	d_{C-H} = 1.09 Å	d_{C-H} = 1.10 Å	-	-	-
	d_{N-C} = 1.52 Å	d_{N-C} = 1.52 Å	d_{N-C} = 1.51 Å			
	d_{C-C} = 1.53 Å	d_{C-C} = 1.53 Å	d_{C-C} = 1.53 Å			

4. Different adsorption configurations of single IL ion-pairs on Na(110) surface

For the ionic liquids PYRFSI and PYRTFSI, we explored several adsorption modes of the ionic pair on the Na(110) surface: (i) configurations with the cation or the anion is oriented vertically toward the surface, denoted cation-Na and anion-Na, respectively, (ii) configurations in which both ions interact simultaneously with the surface in distinct lateral arrangements, referred to as side-on modes. The adsorption geometries are reported in Figure S6, while Figure S7 shows another side-on configuration where the FSI is dissociated (side-on 3: Diss(FSI)).

Energetics, electronic and structural analysis are reported in Table S4 and Table S5.

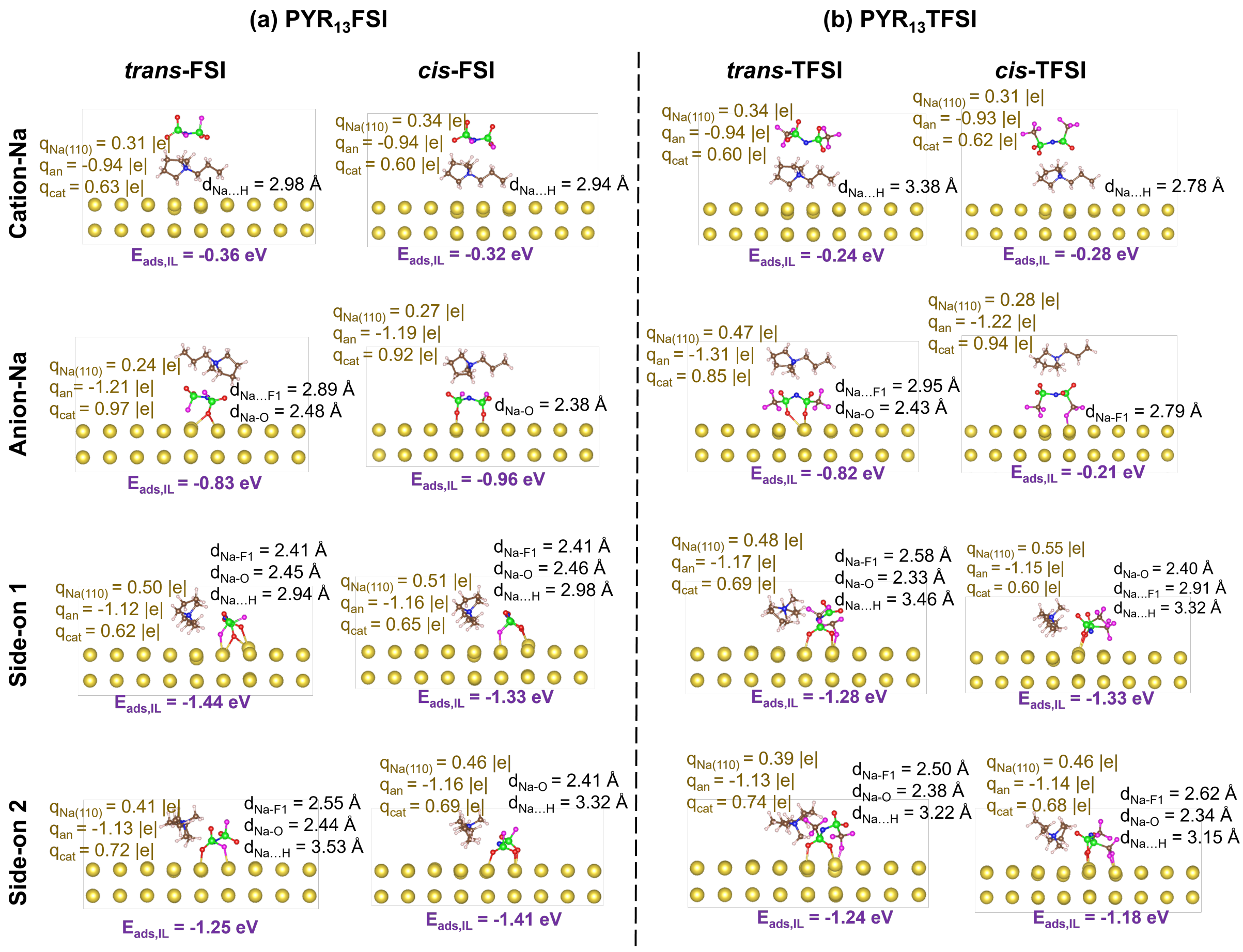

Figure S6. Optimized structures of various adsorption configurations of the ILs with undissociated anions: (a) $PYR_{13}FSI$ and (b) $PYR_{13}TFSI$ on Na(110) surface. The Bader charge-transfer values for Na(110) surface, FSI^- (or $TFSI^-$), and PYR_{13}^+ are denoted as $q_{Na(110)}$, q_{an}, and q_{cat}, respectively. The minimum distances of PYR_{13}^+ and FSI^- (or $TFSI^-$) from Na(110) surface are provided. F1 and F2 represent the undissociated and dissociated F atoms, respectively. Atomic color code: C (brown), N (blue), H (white), S (green), O (red), F (magenta), and Na within Na(110) slab (yellow).

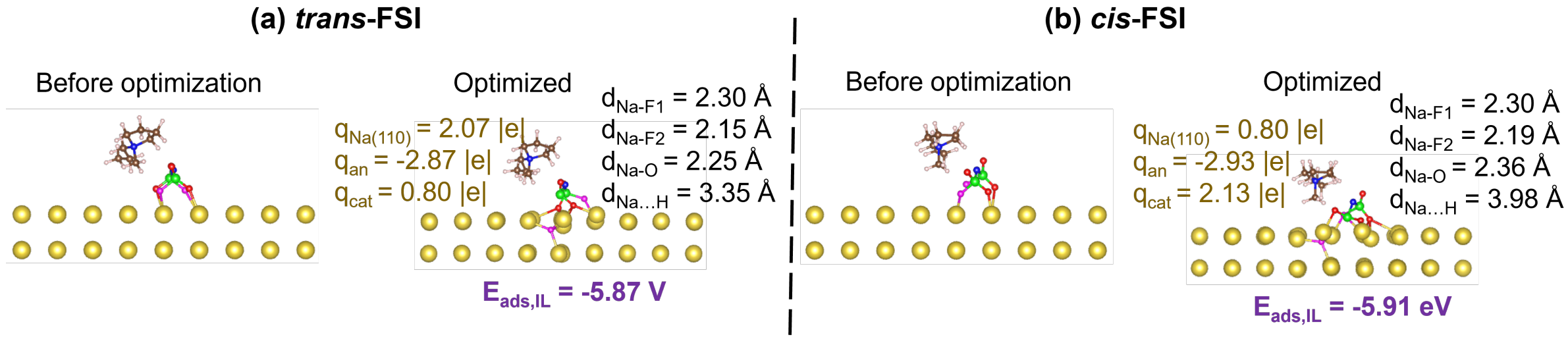

Figure S7. (a) Initial (before optimization) and optimized structures of side-on 3 (Diss(FSI)) configurations of PYR_{13}FSI with (a) *trans*- and and (b) *cis*-FSI, showing the adsorption energies ($E_{ads,IL}$), Bader charge-transfers ($q_{Na(110)}$, q_{an}, and a_{cat}), and minimum distances of PYR_{13} and FSI from Na(110) surface. Atomic color code: C (brown), N (blue), H (white), S (green), O (red), F (magenta), and Na within Na(110) slab (yellow).

Table S4. IL adsorption energies ($E_{ads,IL}$), and the variations in Bader charges at the Na surface ($\Delta q_{Na(110)}$), IL-anion (Δq_{an}), and the IL-cation (Δq_{cat}) after adsorption compared to their isolated states, for different adsorption configurations of IL ion-pairs on Na(110).

PYR_{13}FSI/Na [110]

Adsorption configurations	*trans*-FSI				*cis*-FSI			
	$E_{ads,IL}$ (eV)	$\Delta q_{Na(110)}$ (|e|)	Δq_{an} (|e|)	Δq_{cat} (|e|)	$E_{ads,IL}$ (eV)	$\Delta q_{Na(110)}$ (|e|)	Δq_{an} (|e|)	Δq_{cat} (|e|)
**Cation-Na**	-0.36	0.31	-0.00	-0.31	-0.32	0.34	-0.00	-0.34
**Anion-Na**	-0.83	0.24	-0.27	0.03	-0.96	0.27	-0.24	-0.02
**Side-on 1**	-1.44	0.50	-0.18	-0.32	-1.33	0.51	-0.22	-0.29
**Side-on 2**	-1.25	0.41	-0.19	-0.22	-1.41	0.46	-0.21	-0.25
**Side-on 3**	-5.87	2.07	-1.93	-0.14	-5.91	2.12	-1.98	-0.14

PYR_{13}TFSI/Na [110]

Adsorption configurations	*trans*-TFSI				*cis*-TFSI			
	$E_{ads,IL}$ (eV)	$\Delta q_{Na(110)}$ (|e|)	Δq_{an} (|e|)	Δq_{cat} (|e|)	$E_{ads,IL}$ (eV)	$\Delta q_{Na(110)}$ (|e|)	Δq_{an} (|e|)	Δq_{cat} (|e|)
**Cation-Na**	-0.24	0.34	-0.05	-0.29	-0.28	0.31	0.00	-0.31
**Anion-Na**	-0.82	0.47	-0.43	-0.04	-0.21	0.28	-0.29	0.01
**Side-on 1**	-1.28	0.48	-0.27	-0.21	-1.33	0.55	-0.21	-0.34
**Side-on 2**	-1.24	0.39	-0.24	-0.15	-1.18	0.46	-0.21	-0.26

Table S5. Inter-ionic distances of the IL ion-pairs for different adsorption configurations on Na(110) surface

ILs	Adsorption configurations				
	**Cation-Na**	**Anion-down**	**Side-on 1**	**Side-on 2**	**Side-on 3**
$PYR_{13}FSI$ (*trans*-FSI) *[Fig. 1(a)]*	$d_{F...H}$ = 2.68 Å<br>$d_{O...H}$ = 2.38 Å<br>$d_{N...H}$ = 2.32 Å	$d_{F...H}$ = 2.65 Å<br>$d_{O...H}$ = 2.50 Å<br>$d_{N...H}$ = 2.40 Å	$d_{F...H}$ = 2.73 Å<br>$d_{O...H}$ = 2.58 Å<br>$d_{N...H}$ = 2.51 Å	$d_{F...H}$ = 2.94 Å<br>$d_{O...H}$ = 2.52 Å<br>$d_{N...H}$ = 2.41 Å	$d_{O...H}$ = 2.34 Å<br>$d_{N...H}$ = 2.14 Å
$PYR_{13}FSI$ (*cis*-FSI) *[Fig. S3(a)]*	$d_{F...H}$ = 2.33 Å<br>$d_{O...H}$ = 2.43 Å<br>$d_{N...H}$ = 2.22 Å	$d_{F...H}$ = 2.53 Å<br>$d_{O...H}$ = 2.49 Å<br>$d_{N...H}$ = 2.34 Å	$d_{F...H}$ = 2.69 Å<br>$d_{O...H}$ = 2.76 Å<br>$d_{N...H}$ = 2.59 Å	$d_{F...H}$ = 2.73 Å<br>$d_{O...H}$ = 2.62 Å<br>$d_{N...H}$ = 2.34 Å	$d_{F...H}$ = 2.26 Å<br>$d_{O...H}$ = 2.39 Å<br>$d_{N...H}$ = 2.49 Å
$PYR_{13}TFSI$ (*trans*-TFSI) *[Fig. 1(b)]*	$d_{F...H}$ = 2.47 Å<br>$d_{O...H}$ = 2.30 Å<br>$d_{N...H}$ = 2.27 Å	$d_{F...H}$ = 2.57 Å<br>$d_{O...H}$ = 2.38 Å<br>$d_{N...H}$ = 2.36 Å	$d_{F...H}$ = 2.56 Å<br>$d_{O...H}$ = 2.51 Å<br>$d_{N...H}$ = 2.38 Å	$d_{F...H}$ = 2.60 Å<br>$d_{O...H}$ = 2.57 Å<br>$d_{N...H}$ = 2.56 Å	-
$PYR_{13}TFSI$ (*cis*-TFSI) *[Fig. S3(b)]*	$d_{O...H}$ = 2.34 Å<br>$d_{N...H}$ = 2.39 Å	$d_{O...H}$ = 2.18 Å<br>$d_{N...H}$ = 2.39 Å	$d_{O...H}$ = 2.38 Å<br>$d_{N...H}$ = 3.08 Å	$d_{O...H}$ = 2.32 Å<br>$d_{N...H}$ = 2.56 Å	-

5. Different adsorption configurations of single salt ion-pairs on Na(110) surface

For the salt NaFSI and NaTFSI, we explored several adsorption modes of the ionic pair on the Na(110) surface: (i) configurations with the cation is oriented vertically toward the surface, denoted cation-Na, respectively, (ii) configurations in which both ions interact simultaneously with the surface in distinct lateral arrangements, referred to as side-on modes. The adsorption geometry are reported in Figure S8, while energetics, electronic and structural analysis are reported in Table S6 and Table S7.

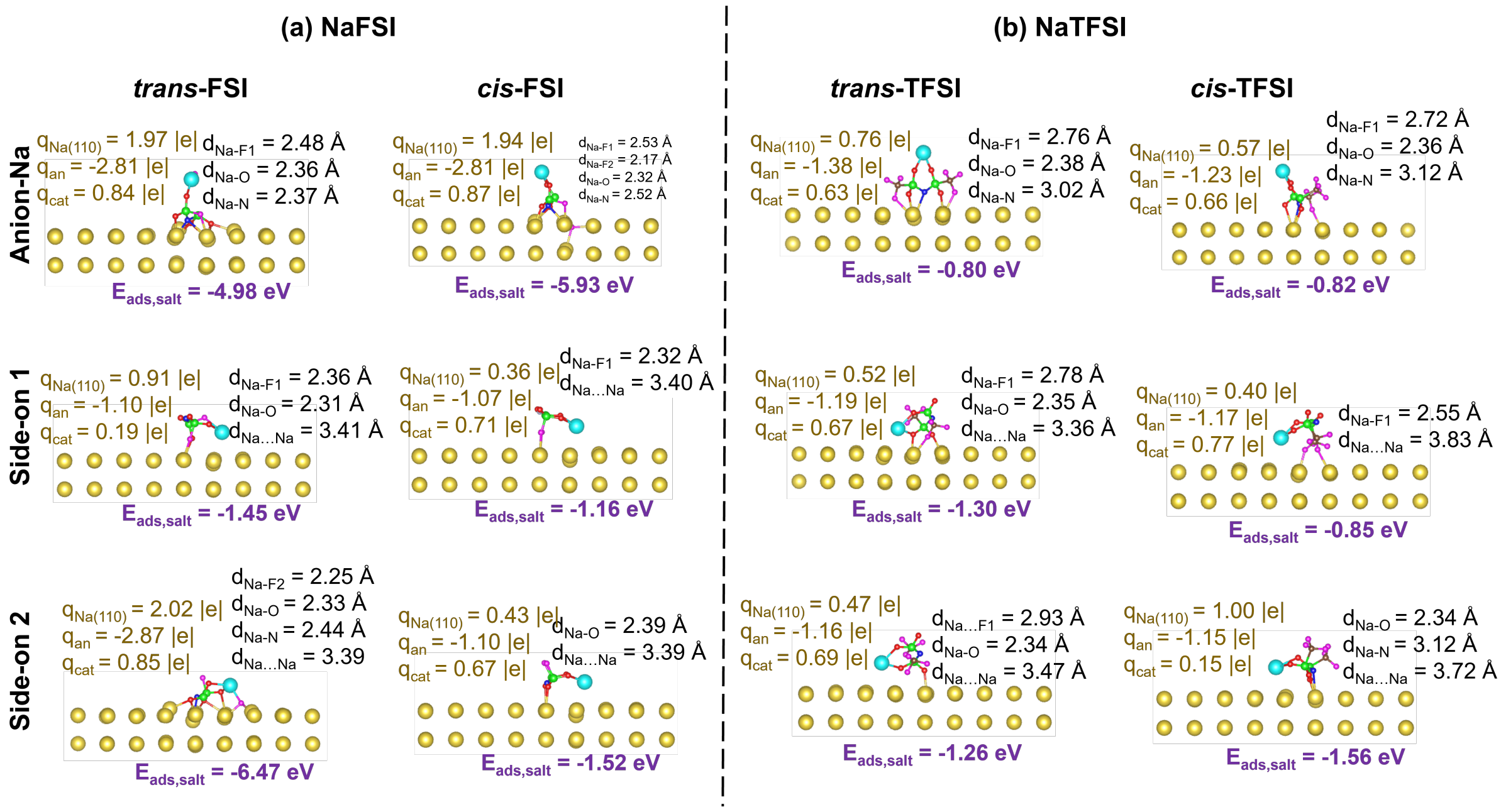

Figure S8. Optimized structures of various adsorption configurations of the salts: (a) NaFSI and (b) NaTFSI on Na(110) surface. The Bader charge-transfer values for Na(110) surface, FSI^- (or $TFSI^-$), and Na^+ are denoted as q_{Na110}, q_{an}, and q_{cat}, respectively. The minimum distances of Na^+ and FSI^- (or $TFSI^-$) from Na(110) surface are provided. F1 and F2 represent the undissociated and dissociated F atoms, respectively. Atomic color code: C (brown), N (blue), H (white), S (green), O (red), F (magenta), Na within Na(110) slab (yellow), and Na within salt (cyan).

Table S6. Salt adsorption energies ($E_{ads,salt}$), and the variations in Bader charges at the Na surface (Δq_{Na}), salt-anion (Δq_{an}), and the Na^+ (Δq_{cat}) after adsorption compared to their isolated states, for different adsorption configurations of salt ion-pairs on Na(110).

NaFSI/Na [110]

Adsorption configurations	*trans*-FSI				*cis*-FSI			
	$E_{ads,salt}$ (eV)	Δq_{Na110} (|e|)	Δq_{an} (|e|)	Δq_{cat} (|e|)	$E_{ads,salt}$ (eV)	Δq_{Na110} (|e|)	Δq_{an} (|e|)	Δq_{cat} (|e|)
**Anion-Na**	-4.98	1.97	-1.91	-0.06	-5.93	1.94	-1.92	0.02
**Side-on 1**	-1.45	0.91	-0.71	-0.20	-1.16	0.36	-0.17	-0.19
**Side-on 2**	-6.47	2.02	-1.97	-0.05	-1.52	0.43	-0.21	-0.22

NaTFSI /Na [110]

Adsorption configurations	*trans*-TFSI				*cis*-TFSI			
	$E_{ads,salt}$ (eV)	Δq_{Na110} (|e|)	Δq_{an} (|e|)	Δq_{cat} (|e|)	$E_{ads,salt}$ (eV)	Δq_{Na110} (|e|)	Δq_{an} (|e|)	Δq_{cat} (|e|)
**Anion-Na**	-0.80	0.76	-0.49	-0.27	-0.82	0.57	-0.34	-0.23
**Side-on 1**	-1.30	0.52	-0.30	-0.22	-0.85	0.40	-0.28	-0.12
**Side-on 2**	-1.26	0.47	-0.27	-0.20	-1.56	1.00	-0.26	-0.74

Table S7. Inter-ionic distances of the salt ion-pairs for different adsorption configurations on Na(110) surface

**Salts**	**Adsorption configurations**		
	**Anion-Na**	**Side-on 1**	**Side-on 2**
NaFSI (*trans*-FSI) *[isolated: Figure 1(c)]*	$d_{Na\text{-}O}$ = 2.23 Å<br>$d_{Na\text{-}F}$ = 1.96 Å	$d_{Na\text{-}O}$ = 2.32 Å<br>$d_{Na\text{-}F}$ = 2.49 Å	$d_{Na\text{-}O}$ = 2.29 Å<br>$d_{Na\text{-}F}$ = 2.16 Å
NaFSI (*cis*-FSI) *[isolated: Figure S3(c)]*	$d_{Na\text{-}O}$ = 2.13 Å	$d_{Na\text{-}O}$ = 2.32 Å	$d_{Na\text{-}O}$ = 2.35 Å
NaTFSI (*trans*-TFSI) *[isolated: Figure 1(d)]*	$d_{Na\text{-}O}$ = 2.30 Å	$d_{Na\text{-}O}$ = 2.28 Å	$d_{Na\text{-}O}$ = 2.32 Å
NaTFSI (*cis*-TFSI) *[isolated: Figure S3(d)]*	$d_{Na\text{-}O}$ = 2.27 Å	$d_{Na\text{-}O}$ = 2.28 Å	$d_{Na\text{-}O}$ = 2.34 Å

6. Dynamics of electrolytes on Na(110) surface

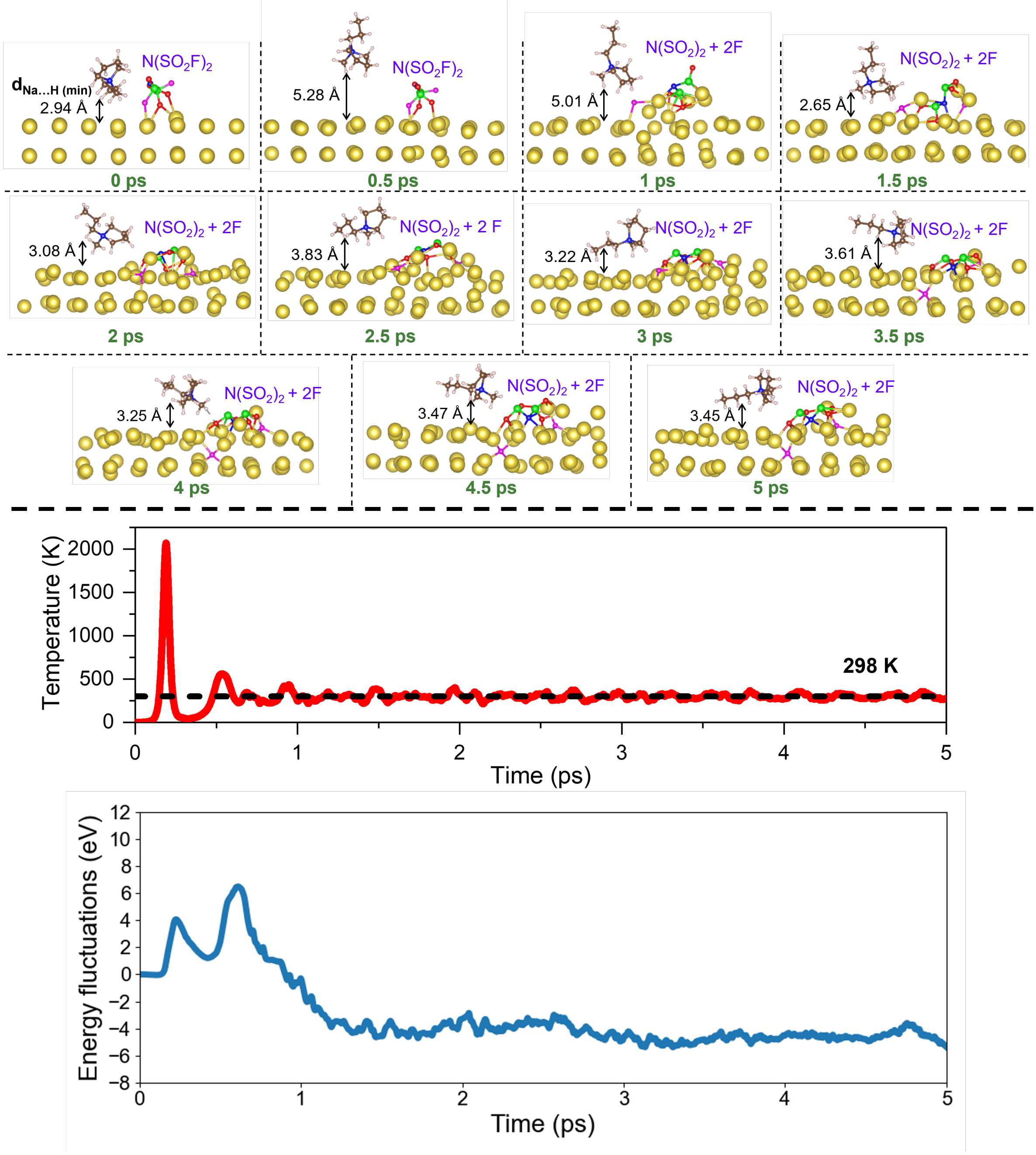

Figure S9. Upper panel: Complete trajectories of FSI decomposition for $PYR_{13}FSI$ with undissociated FSI initial state. Lower panel: Corresponding Temperature vs Time (red) and total Energy fluctuations vs Time (blue) profiles. Atomic color code: C (brown), N (blue), H (white), S (green), O (red), F (magenta), and Na within Na(110)slab (yellow).

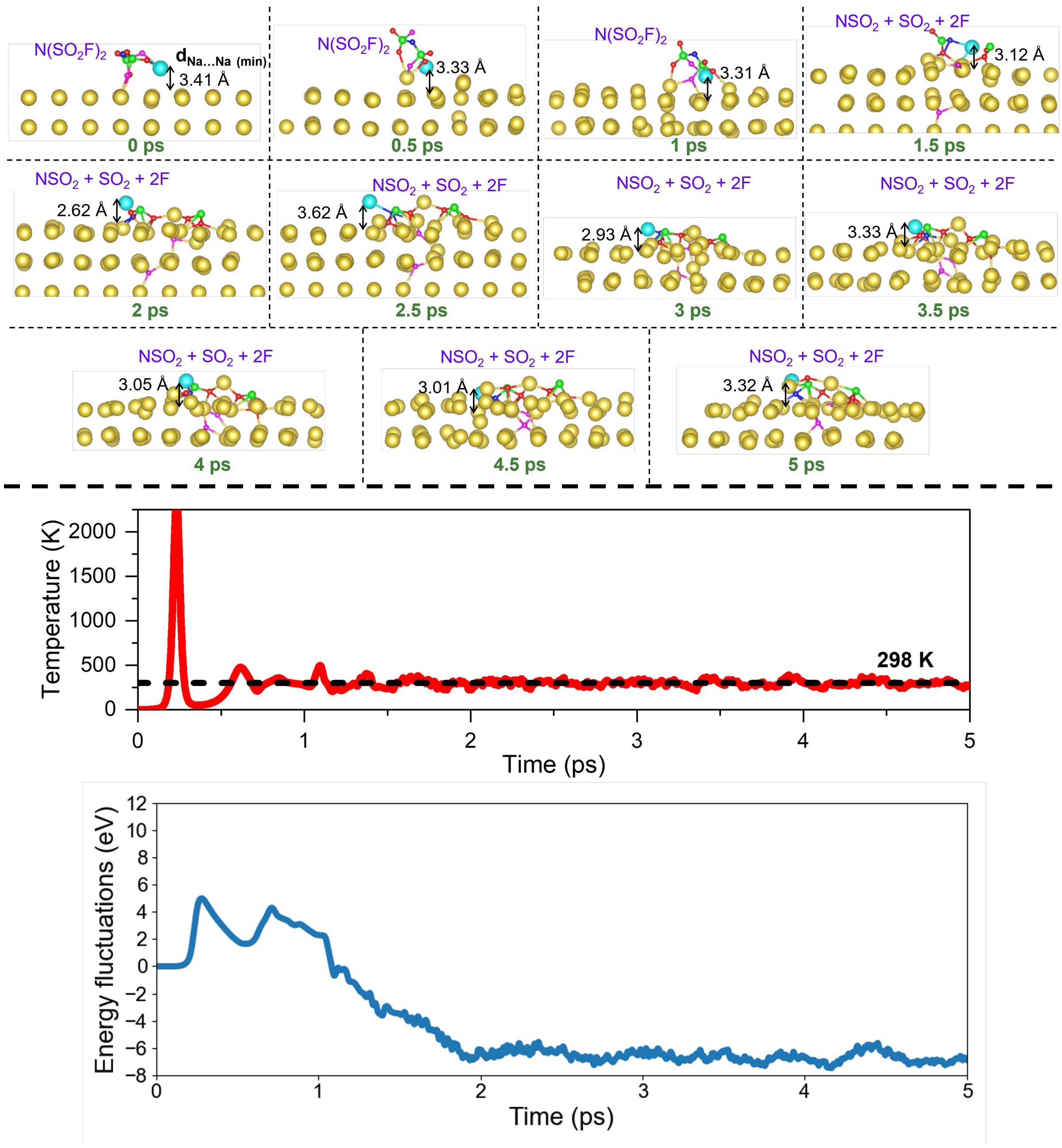

Figure S10. Upper panel: Complete trajectories of FSI decomposition for NaFSI with undissociated FSI initial state (upper panel). Lower panel: Corresponding Temperature vs Time (red) and total Energy fluctuations vs Time (blue) profiles. Atomic color code: C (brown), N (blue), H (white), S (green), O (red), F (magenta), Na within Na(110) slab (yellow), and Na within salt (cyan).

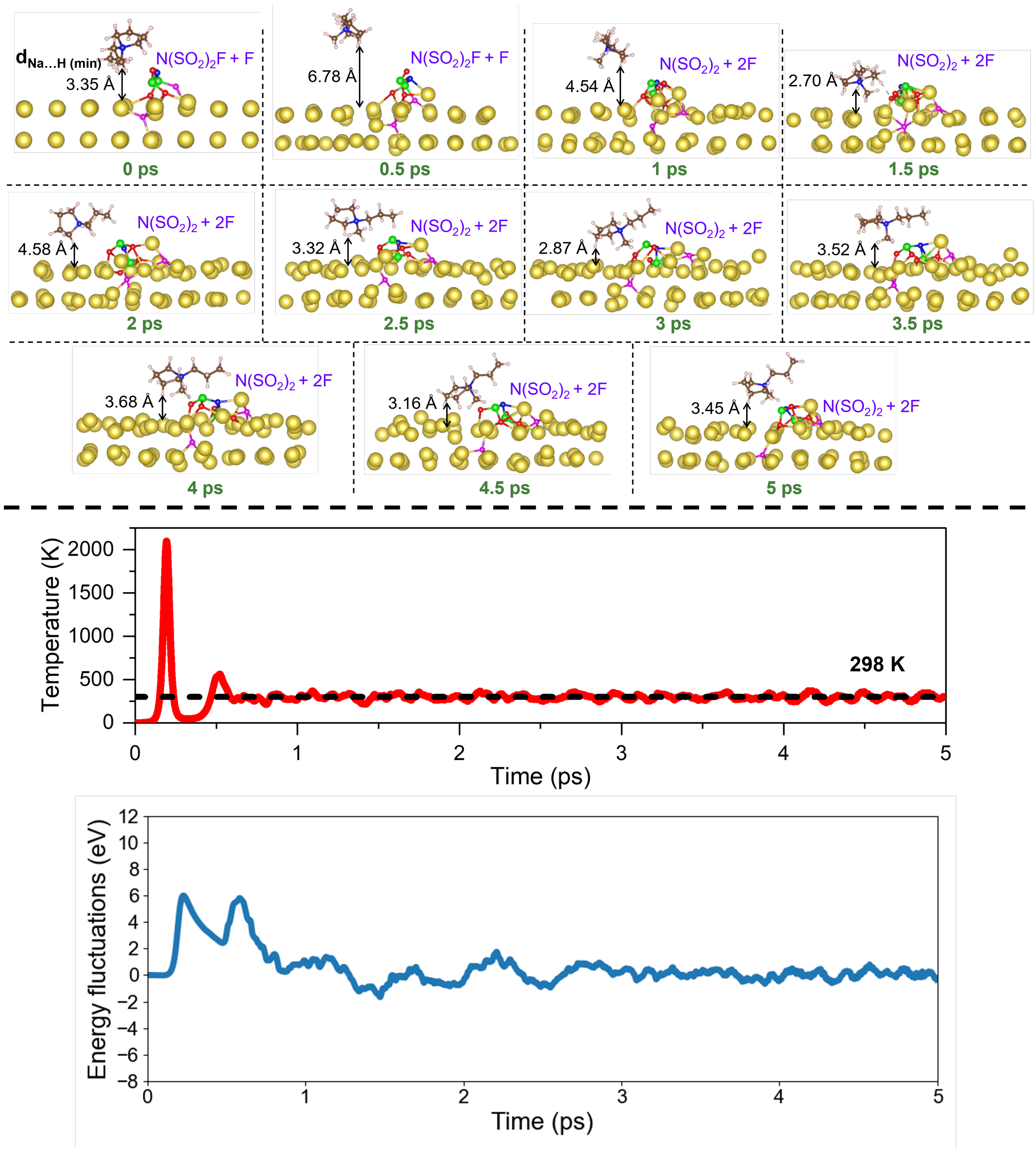

Figure S11. Upper panel: Complete trajectories of FSI decomposition for $PYR_{13}FSI$ with dissociated FSI initial state (upper panel). Lower panel: Corresponding Temperature vs Time (red) and total Energy fluctuations vs Time (blue) profiles. Atomic color code: C (brown), N (blue), H (white), S (green), O (red), F (magenta), and Na within Na(110) slab (yellow).

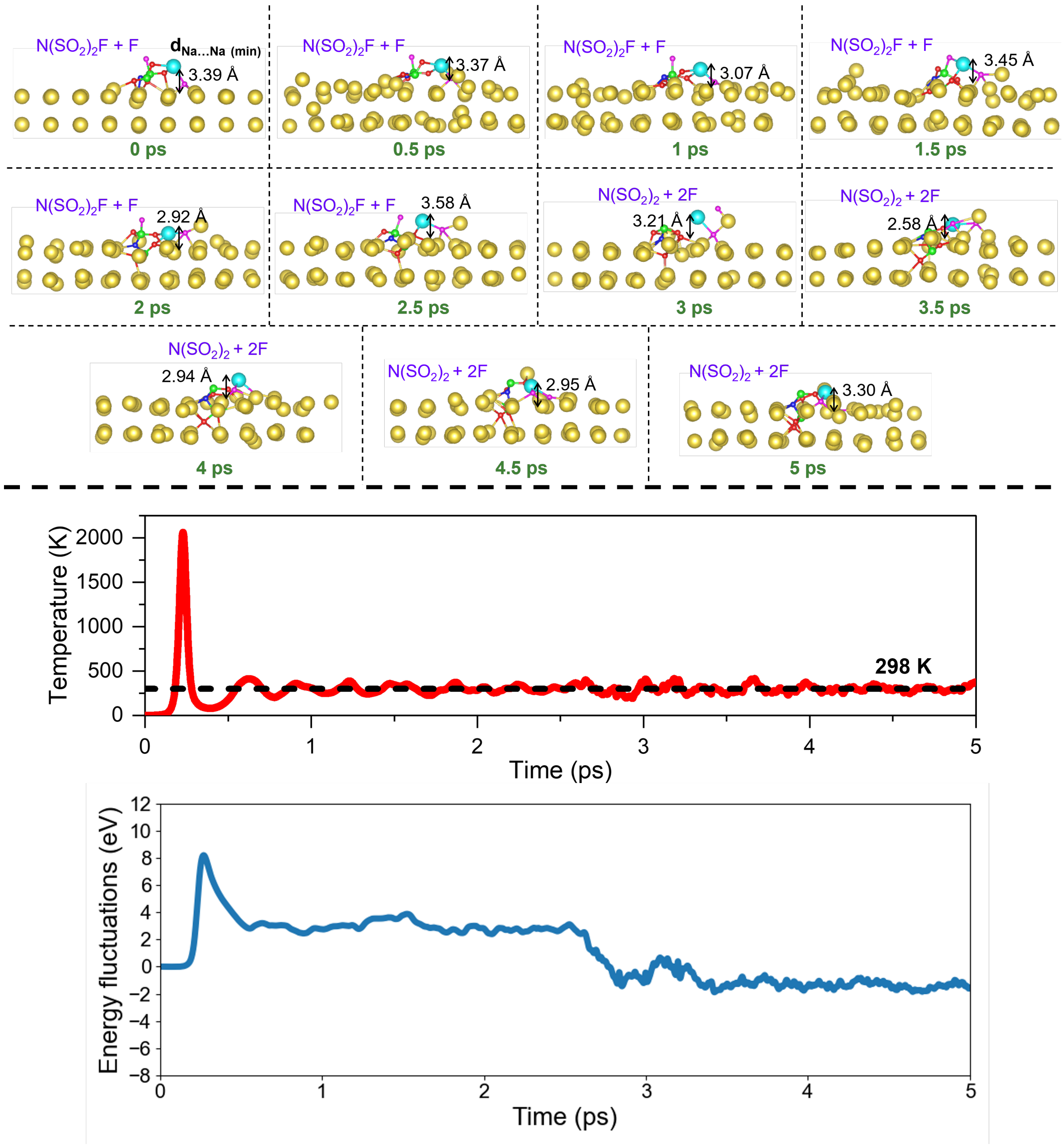

Figure S12. Upper panel: Complete trajectories of FSI decomposition for NaFSI with dissociated FSI initial state (upper panel). Lower panel: Corresponding Temperature vs Time (red) and total Energy fluctuations vs Time (blue) profiles. Atomic color code: C (brown), N (blue), H (white), S (green), O (red), F (magenta), Na within Na(110) slab (yellow), and Na within salt (cyan).

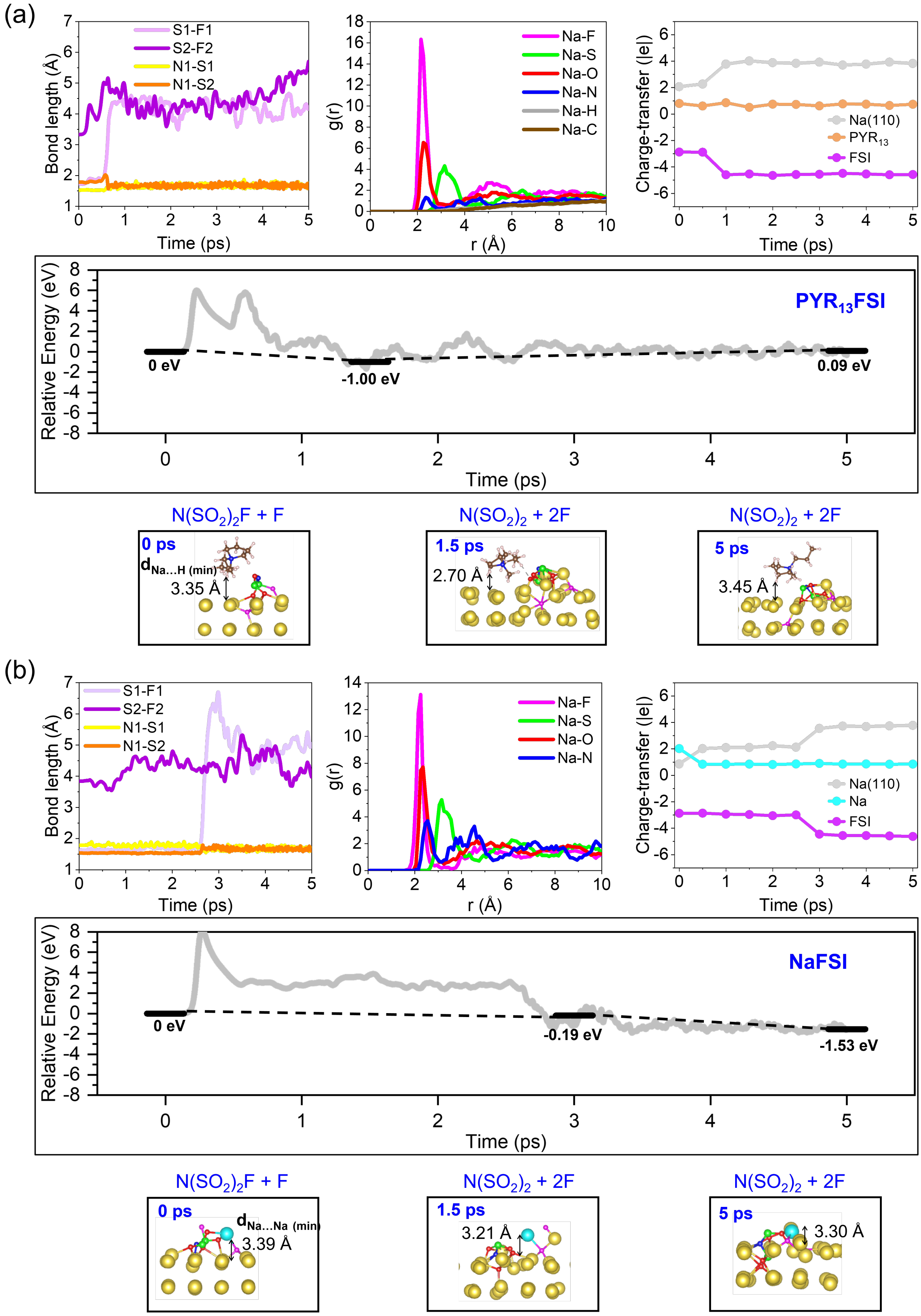

Figure S13. AIMD simulation illustrating the FSI decomposition pathway for (a) $PYR_{13}FSI$ with dissociated FSI initial state (ref. Fig. 2c) – Upper panel: bond-length evolution with time (left), RDF plots showing

coordination between Na atoms in Na(110) slab and atoms in PYR_{13} or decomposed FSI (middle), and Bader charge-transfer evolution with time (right); lower panel: Energetics and snapshots showing the primary decomposition steps with the distance between PYR_{13} and Na(110) surface ($d_{Na...H\ (min)}$); Electronic energies extracted from AIMD trajectories are shown in grey color in the background, (b) NaFSI with dissociated FSI initial state (ref. Fig. 3c) – Upper panel: bond-length evolution with time (left), RDF plots showing coordination between Na atoms in Na(110) slab and atoms in decomposed FSI (middle), and Bader charge-transfer evolution with time (right); lower panel: Energetics and snapshots showing the primary decomposition steps with distance between Na atom in salt and Na(110) surface ($d_{Na...Na\ (min)}$); Electronic energies extracted from AIMD trajectories are shown in grey color in the background. Atomic color code: C (brown), N (blue), H (white), S (green), O (red), F (magenta), Na within Na(110) slab (yellow), and Na within salt (cyan).

Table S8. Snapshots of the final decomposition states of FSI anions in $PYR_{13}FSI$ and NaFSI

IL/Salt	AIMD starting point	Final decomposed structure	Bond lengths
$PYR_{13}FSI$	$[PYR_{13}]$ (ads) + $N(SO_2F)_2$ (ads)	**t = 5 ps**<br>$[PYR_{13}]$ (ads) + $N(SO_2)_2$ (ads) + 2F (ads)<br>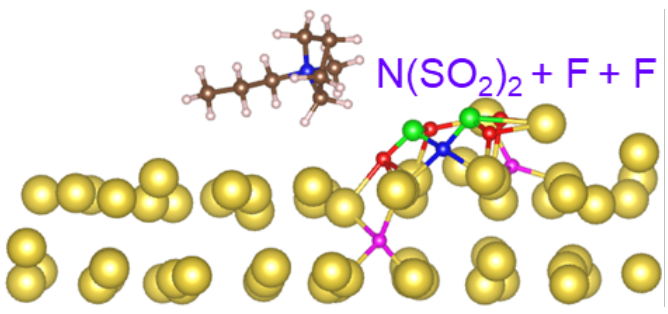	$d_{Na\text{-}F}$ = 2.06 – 2.47 Å<br>$d_{Na\text{-}O}$ = 2.19 – 2.75 Å<br>$d_{Na\text{-}S}$ = 2.93 – 3.16 Å<br>$d_{Na\text{-}N}$ = 2.46 Å<br>$d_{Na...H}$ = 4.02 Å
	$[PYR_{13}]$ (ads) + $N(SO_2)_2F$ (ads) + F (ads)	**t = 5 ps**<br>$[PYR_{13}]$ (ads) + $N(SO_2)_2$ (ads) + 2F (ads)<br>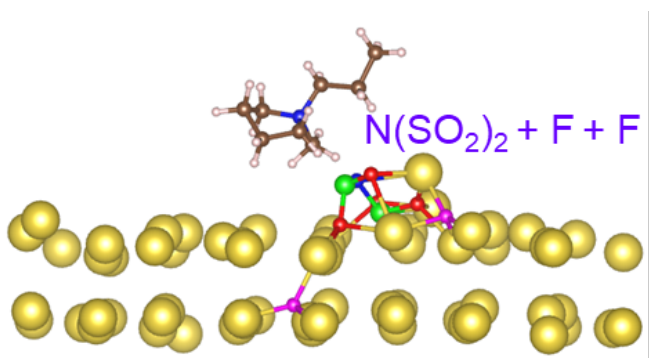	$d_{Na\text{-}F}$ = 2.09 – 2.38 Å<br>$d_{Na\text{-}O}$ = 2.13 – 2.88 Å<br>$d_{Na\text{-}S}$ = 2.84 – 3.18 Å<br>$d_{Na\text{-}N}$ = 3.08 Å<br>$d_{Na...H}$ = 3.45 Å
	$[PYR_{13}]$ (ads) + $N(SO_2)_2$ (ads) + 2F (ads)	**t = 10 ps**<br>$[PYR_{13}]$ (ads) + $N(SO_2)_2$ (ads) + 2F (ads)<br>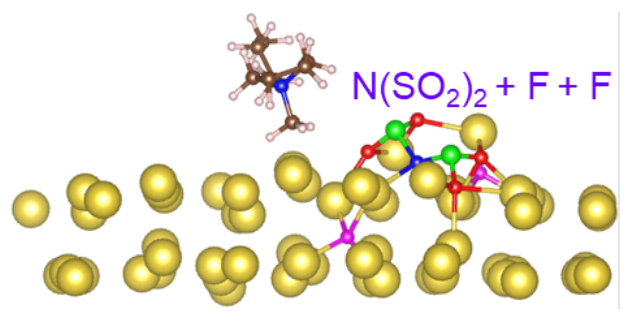	$d_{Na\text{-}F}$ = 1.96 – 2.46 Å<br>$d_{Na\text{-}O}$ = 2.31 – 2.58 Å<br>$d_{Na\text{-}S}$ = 3.12 – 3.34 Å<br>$d_{Na\text{-}N}$ = 2.46 – 2.70 Å<br>$d_{Na...H}$ = 3.19 Å

NaFSI	Na (ads) + $N(SO_2)_2F_2$ (ads)	**t = 5 ps**<br>$[PYR_{13}]$ (ads) + $N(SO_2)$ (ads) + SO_2 (ads) + 2F (ads)<br>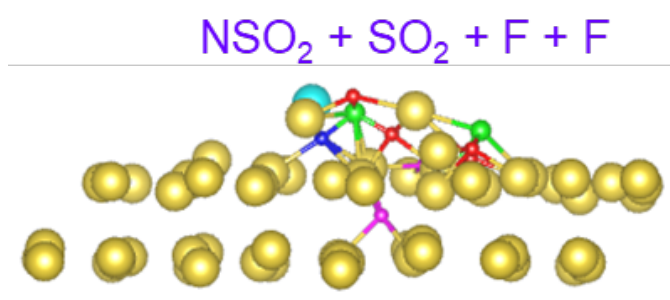 	$d_{Na\text{-}F}$ = 2.17 – 2.44 Å<br>$d_{Na\text{-}O}$ = 2.18 – 2.53 Å<br>$d_{Na\text{-}S}$ = 2.68 – 2.94 Å<br>$d_{Na\text{-}N}$ = 2.37 – 2.65 Å<br>$d_{Na...Na}$ = 3.32 Å
	Na (ads) + $N(SO_2)_2F$ (ads) + F (ads)	**t = 5 ps**<br>$[PYR_{13}]$ (ads) + $N(SO_2)_2$ (ads) + 2F (ads)<br>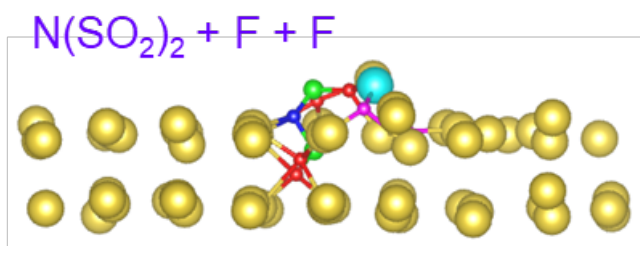 	$d_{Na\text{-}F}$ = 2.04 – 2.49 Å<br>$d_{Na\text{-}O}$ = 2.22 – 2.49 Å<br>$d_{Na\text{-}S}$ = 2.97 – 3.19 Å<br>$d_{Na\text{-}N}$ = 2.54 – 2.58 Å<br>$d_{Na...Na}$ = 3.30 Å

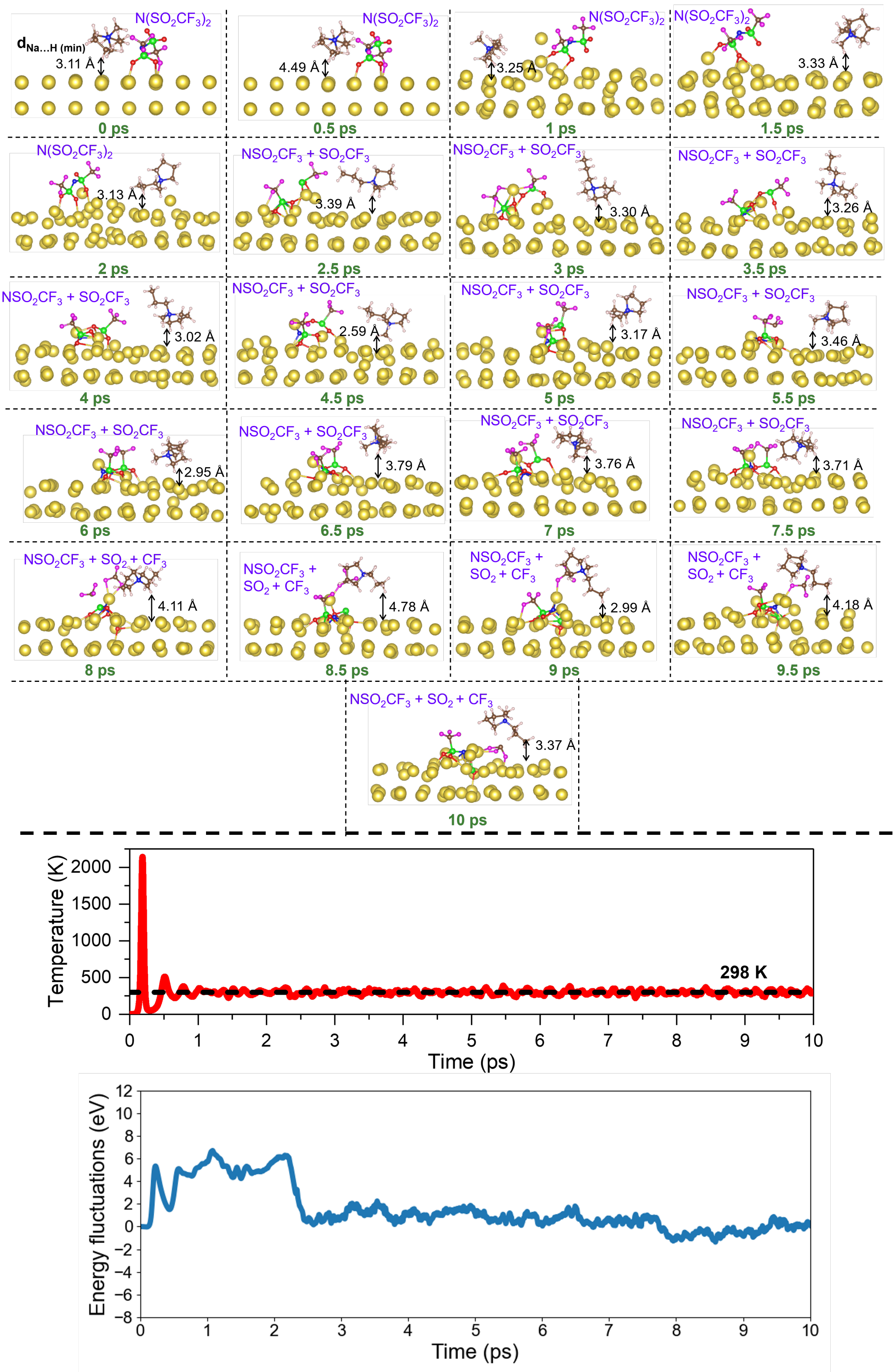

Figure S14. Upper panel: Complete trajectories of FSI decomposition for $PYR_{13}TFSI$ (upper panel). Lower panel: Corresponding Temperature vs Time profile (red) and *total* Energy fluctuations vs Time (blue) profiles. Atomic color code: C (brown), N (blue), H (white), S (green), O (red), F (magenta), and Na within Na(110) slab (yellow).

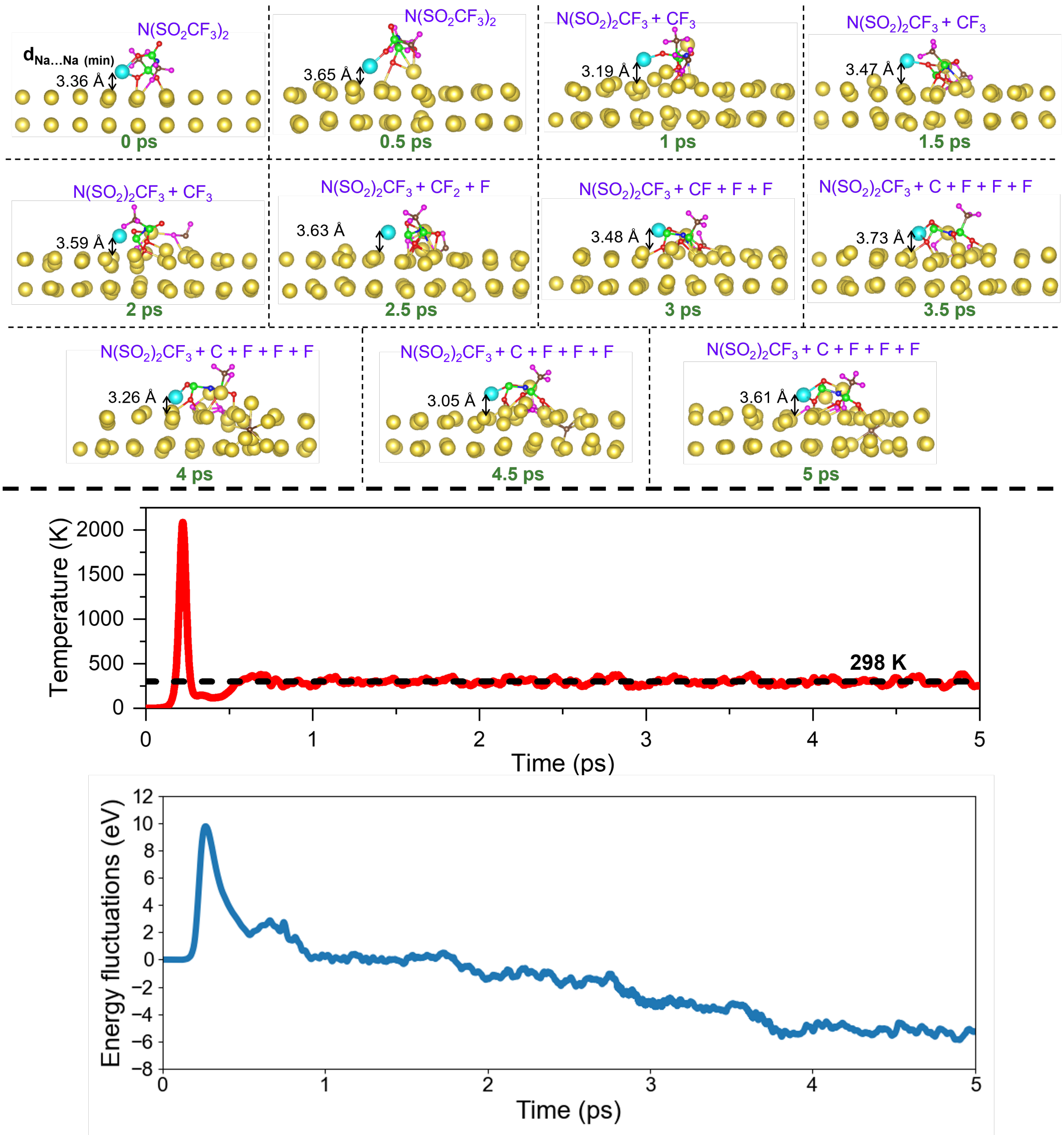

Figure S15. Upper panel: Complete trajectories of FSI decomposition for NaTFSI (upper panel). Lower panel: Corresponding Temperature vs Time profile (red) and total Energy fluctuations vs Time (blue) profiles. Atomic color code: C (brown), N (blue), H (white), S (green), O (red), F (magenta), Na within Na(110) slab (yellow), and Na within salt (cyan).

Table S9. Snapshots of the final decomposition states of TFSI anions in $PYR_{13}TFSI$ and NaTFSI

IL/Salt	AIMD starting point	Final decomposed structure	Bond lengths
$PYR_{13}TFSI$	$[PYR_{13}]$ (ads) + $N(SO_2CF_3)_2$ (ads)	**t = 5 ps**<br>$[PYR_{13}]$ (ads) + NSO_2CF_3 (ads) + CF_3 (ads)<br>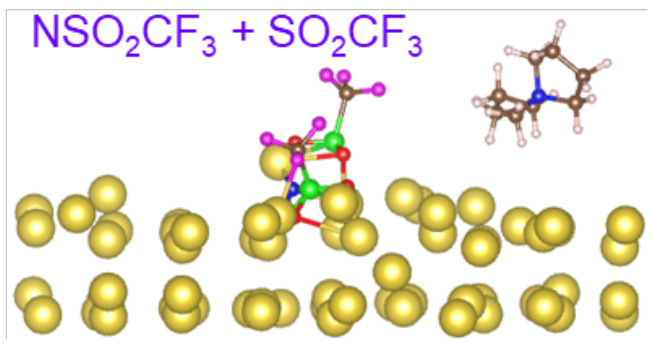 	$d_{Na\text{-}F}$ = 2.41 Å<br>$d_{Na\text{-}O}$ = 2.06 – 2.59 Å<br>$d_{Na\text{-}S}$ = 2.73 – 3.18 Å<br>$d_{Na\text{-}N}$ = 2.32-2.40 Å<br>$d_{Na...H}$ = 3.17 Å
	$[PYR_{13}]$ (ads) + NSO_2CF_3 (ads) + CF_3 (ads)	**t = 10 ps**<br>$[PYR_{13}]$ (ads) + $N(SO_2)_2$ (ads) + 2F (ads)<br>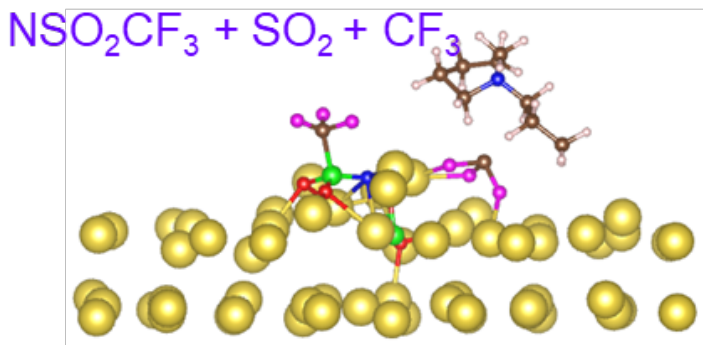 	$d_{Na\text{-}F}$ = 2.25 – 2.34 Å<br>$d_{Na\text{-}O}$ = 2.08 – 2.49 Å<br>$d_{Na\text{-}S}$ = 2.79 – 2.81 Å<br>$d_{Na\text{-}N}$ = 2.31-3.01 Å<br>$d_{Na...H}$ = 3.37 Å
NaTFSI	$[PYR_{13}]$ (ads) + $N(SO_2CF_3)_2$ (ads)	**t = 5 ps**<br>$[PYR_{13}]$ (ads) + $N(SO_2)_2CF_3$ (ads) + C (ads) + 3F (ads)<br>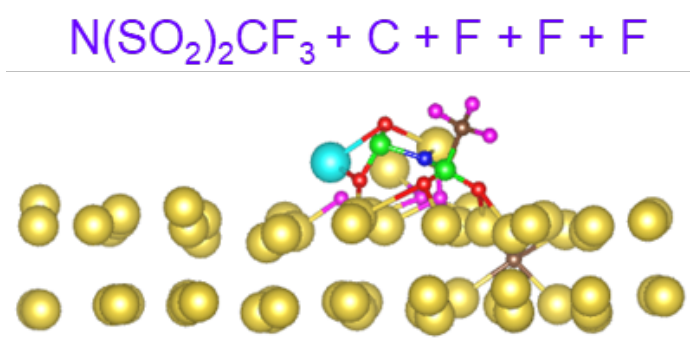 	$d_{Na\text{-}F}$ = 2.02 – 2.50 Å<br>$d_{Na\text{-}O}$ = 2.19 – 2.93 Å<br>$d_{Na\text{-}S}$ = 2.95 Å<br>$d_{Na\text{-}N}$ = 2.54 Å<br>$d_{Na...Na}$ = 3.61 Å

References

(1) Gaissmaier, D.; van den Borg, M.; Fantauzzi, D.; Jacob, T. Microscopic Properties of Na and Li—A First Principle Study of Metal Battery Anode Materials. *ChemSusChem* **2020**, *13* (4), 771–783. https://doi.org/10.1002/cssc.201902860.

(2) Zeng, Y.; Zhang, S.; Yao, Y.; Liu, J.; Zhou, N.; Tang, J.; Zhang, K.; Li, Y.; Hu, J.; Yang, B.; Peng, C.; Liu, H. Structure and Interaction of Ionic Liquid Monolayer on Lithium from First-Principles. *Journal of Physical Chemistry C* **2024**, *128* (39), 16766–16780. https://doi.org/10.1021/acs.jpcc.4c04828.
